# Biochemical and Phylogenetic Characterization of a Novel NADP^+^-Specific Isocitrate Dehydrogenase From the Marine Microalga *Phaeodactylum tricornutum*

**DOI:** 10.3389/fmolb.2021.702083

**Published:** 2021-07-05

**Authors:** Shiping Huang, Jiaxin Zhao, Wenjing Li, Peng Wang, Zhenglian Xue, Guoping Zhu

**Affiliations:** ^1^ Anhui Provincial Key Laboratory of Molecular Enzymology and Mechanism of Major Diseases, Key Laboratory of Biomedicine in Gene Diseases and Health of Anhui Higher Education Institutes, College of Life Sciences, Anhui Normal University, Wuhu, China; ^2^ College of Biological and Food Engineering, Anhui Polytechnic University, Wuhu, China

**Keywords:** isocitrate dehydrogenase, *Phaeodactylum tricornutum*, biochemical characterization, enzymology, coenzyme specificity determinants, thermostability

## Abstract

Isocitrate dehydrogenase (IDH) family of proteins is classified into three subfamilies, namely, types I, II, and III. Although IDHs are widely distributed in bacteria, archaea, and eukaryotes, all type III IDHs reported to date are found only in prokaryotes. Herein, a novel type III IDH subfamily member from the marine microalga *Phaeodactylum tricornutum* (PtIDH2) was overexpressed, purified, and characterized in detail for the first time. Relatively few eukaryotic genomes encode this type of IDH and PtIDH2 shares the highest homology with marine bacterial monomeric IDHs, suggesting that PtIDH2 originated through a horizontal gene transfer event between a marine alga and a bacterium. Size-exclusion chromatography revealed that the native PtIDH2 is a homotetramer (∼320 kDa) in solution, comprising four monomeric IDH-like subunits (80 kDa each). Enzymatic characterization showed that PtIDH2 is a bivalent metal ion-dependent enzyme and Mn^2+^ is the optimal activator. The recombinant PtIDH2 protein exhibited maximal activity at 35°C and pH 8.0 in the presence of Mn^2+^. Heat-inactivation analysis revealed that PtIDH2 is a cold-adapted enzyme. Kinetic analysis demonstrated that PtIDH2 is a completely NADP^+^-specific IDH with no detectable NAD^+^-associated catalytic activity. The three putative key NADP^+^-binding residues (His604, Arg615, and Arg664) in PtIDH2 were also evaluated by site-directed mutagenesis. The H^604^L/R^615^D/R^664^S triple mutant showed a 3.25-fold preference for NAD^+^ over NADP^+^, implying that the coenzyme specificity of PtIDH2 can be converted from NADP^+^ to NAD^+^ through rational engineering approaches. Additionally, the roles of the conserved residues Ala718 and Leu742 in the thermostability of PtIDH2 were also explored by site-directed mutagenesis. We found that the L^742^F mutant displayed higher thermostability than wild-type PtIDH2. This study expands the phylogeny of the IDH family and provides new insights into the evolution of IDHs.

## Introduction

Isocitrate dehydrogenase (IDH) plays a vital role in the tricarboxylic acid (TCA) cycle (also known as the Krebs cycle or citric acid cycle), catalyzing the oxidative decarboxylation of isocitrate into α-ketoglutarate (α-KG) and CO_2_ using NAD^+^ or NADP^+^ as a coenzyme. Based on their cofactor specificity, IDHs can be classified as either NAD^+^-specific IDH (EC 1.1.1.41, NAD-IDH) or NADP^+^-specific IDH (EC 1.1.1.42, NADP-IDH). NAD-IDH catalysis generates NADH for ATP production and energy metabolism, while NADP-IDH catalysis generates NADPH, an important source of reducing power necessary for the biosynthesis of cellular components and defenses against oxidative damage (Jo et al., 2001; Sun and Park, 2003; Wang et al., 2015). Mutations in human cytosolic and mitochondrial NADP-IDH (IDH1 and IDH2, respectively) are frequently identified in a variety of human cancers, such as glioblastoma (GBM) and angioimmunoblastic T cell lymphoma (AITL) (Waitkus et al., 2018; Eniafe and Jiang, 2021). Several studies have reported that IDHs with a mutation in a conserved arginine (Arg132 of IDH1 and Arg172 of IDH2) residue present in the substrate-binding site acquire a novel function, i.e., they catalyze the production of D-2-hydroxyglutarate (D-2HG) from α-KG. D-2HG is an oncometabolite that promotes tumorigenesis *via* the reprogramming of DNA and histone methylation (Kohanbash et al., 2017; Sulkowski et al., 2020). These observations highlight that IDHs are not only key metabolic enzymes but are also important clinical diagnostic biomarkers for certain tumors as well as potential targets for therapeutic drugs.

Based on subunit composition, IDHs can be categorized into five types: monomers, homodimers, homotetramers, homohexamers, and heterooligomers. However, differences among IDHs are far greater when sources and coenzyme specificity are also considered. Phylogenetic analysis led to the identification of three IDH protein subfamilies, namely types I, II, and III (Wang et al., 2015). Type I IDHs comprise bacterial and archaeal homodimeric NAD(P)-IDHs, bacterial homotetrameric NAD-IDHs, and eukaryotic mitochondrial heterooligomeric NAD-IDHs; type II IDHs are mainly comprised of bacterial homodimeric NADP-IDHs, bacterial homohexameric NAD-IDHs, and eukaryotic mitochondrial and cytoplasmic homodimeric NADP-IDHs; meanwhile, type III IDHs encompass all prokaryotic monomeric NAD(P)-IDHs. Notably, we recently reported a group of homodimeric NADP-IDHs from pathogenic bacteria, such as *Acinetobacter baumannii*, that contain two monomeric IDH-like subunits and belong to the type III IDH subfamily (Wang et al., 2018). Although type I and II IDHs display similar subunit molecular mass (40–45 kDa) and three-dimensional structures, their amino acid sequence identities are very low (<15%). Furthermore, type III IDH subfamily members have longer polypeptide chains (∼740 amino acids) and low sequence homology (<10%) with type I and II IDHs, suggesting that they evolved independently.

Studies have demonstrated that the NAD^+^-specific phenotype is an ancestral trait and that NADP^+^-specific bacterial IDHs arose at the same time as eukaryotic mitochondria, approximately 3.5 billion years ago (Zhu et al., 2005). The switch in coenzyme specificity from NAD^+^ to NADP^+^ by IDHs is likely to have been an ancient adaptive evolutionary event that allowed bacteria to survive on energy-poor compounds such as acetate and other two-carbon resources. This hypothesis has been verified by reverse evolution experiments using typical bacterial IDHs, including those from *Escherichia coli* (EcIDH, type I NADP-IDH subfamily) (Hurley et al., 1996), *Bifidobacterium longum* (BlIDH, type II NADP-IDH) (Huang et al., 2016), and *Campylobacter* sp. (CaIDH, type III NAD-IDH) (Wang et al., 2015). However, information pertaining to eukaryotic IDHs is scarce, and it remains unknown whether similar evolutionary mechanisms of coenzyme specificity exist in eukaryotic IDHs.

IDHs are widely distributed in all living organisms, including archaea, bacteria, and eukaryotes. However, to date, type III IDHs, such as *Azotobacter vinelandii* IDH (AvIDH), *Corynebacterium glutamicum* IDH (CgIDH), *Campylobacter concisus* IDH (Wang et al., 2019), and *Methanococcoides methylutens* IDH (MmIDH) (Wang et al., 2020), have been identified only in prokaryotes. Over recent years, with the development of high-throughput genome sequencing technology, a large number of complete genome sequences from marine organisms have become available. Additionally, the increased characterization of IDH family members has allowed for the expansion and refinement of the classification of the IDH family of proteins. Using bioinformatic analysis, we have found that, in addition to typical type II homodimer NAD^+^-specific IDHs (Tang et al., 2015; Huang et al., 2020), eukaryotes further contain a class of putative type III NADP^+^-specific IDHs, represented by the potential monomeric NADP-IDH from *Phaeodactylum tricornutum*, a unicellular marine eukaryotic microalga.

*P. tricornutum*, a model diatom species, is commonly found in marine ecosystems and plays a significant role in carbon fixation and the global mineral cycle (Yao et al., 2014). Furthermore, *P. tricornutum* has shown great potential for application in animal feed and human nutrition, especially because of its fast growth rate and high lipid production. Analysis of the *P. tricornutum* genome has revealed several unique characteristics, including the presence of hundreds of genes transferred from bacteria, suggesting that *P. tricornutum* has a complicated evolutionary history. We have previously found that *P. tricornutum* contains two putative IDHs, and characterized in detail the biochemical properties and crystal structure of PtIDH1, a type II homodimeric NAD-IDH (Huang et al., 2020). In the current study, the enzymology of a putative type III NADP-IDH from *P. tricornutum* (PtIDH2) was characterized, including its oligomeric form in solution, kinetics, and thermostability, as well as how its activity is affected by pH, temperature, and metal ions. Moreover, through site-directed mutagenesis, we achieved the conversion of the coenzyme specificity of PtIDH2 from NADP^+^ to NAD^+^. The finding of a novel type III eukaryotic NADP-IDH expands the phylogeny of the IDH protein family and provides useful insights into the evolution of the type III IDH subfamily.

## Materials and Methods

### Bioinformatic Analysis

The amino acid sequences of PtIDH2 and other IDHs were obtained from the UniProt database (https://www.uniprot.org/). The X-ray crystallographic structure of AvIDH (PDB ID: 1J1W) was downloaded from the Protein Data Bank archive (https://www.rcsb.org). Protein sequence similarity and identity were analyzed using the BLAST online server (https://blast.ncbi.nlm.nih.gov/Blast.cgi). Structure-based amino acid sequence alignment was conducted using the T-coffee Multiple Sequence Alignment Server (https://tcoffee.vital-it.ch/apps/tcoffee/index.html) and the ESPript web tool (http://espript.ibcp.fr/ESPript/cgi-bin/ESPript.cgi) (Notredame et al., 2000; Robert and Gouet, 2014). Subcellular localization and signal peptide prediction for PtIDH2 was conducted using the Cell-PLoc v.2.0 server (http://www.csbio.sjtu.edu.cn/bioinf/Cell-PLoc-2), the TargetP v.2.0 server (http://www.cbs.dtu.dk/services/TargetP/), the SignalP v.5.0 server (http://www.cbs.dtu.dk/services/SignalP), and WoLF PSORT (https://wolfpsort.hgc.jp/) (Horton et al., 2007; Chou and Shen, 2008; Almagro Armenteros et al., 2019a; Almagro Armenteros et al., 2019b). The phylogenetic tree of the IDH protein family was reconstructed with MEGA7 software (Kumar et al., 2016). According to high similarity, AvIDH (PDB ID: 1ITW) was selected as a template for homology modeling of wild-type PtIDH2, mutational PtIDH2 and *Campylobacter* sp. IDH. Automated structure homology modeling was performed using the Swiss-model server (https://swissmodel.expasy.org) (Waterhouse et al., 2018). Energy minimization and structural analysis of models were done with the UCSF Chimera software (Pettersen et al., 2004). Structure comparison, visualization, and image preparation were conducted using PyMOL and UCSF Chimera software (Pettersen et al., 2004; Bramucci et al., 2012).

### Strains, Plasmids, and Reagents

*E. coli* DH5α cells, *E. coli* Rosetta (DE3) cells, and the pET-28b(+) expression vector were preserved at low temperature in our laboratory. PrimeSTAR Max DNA Polymerase was purchased from TaKaRa (Dalin, China). Restriction enzymes (NdeI and XhoI), T4 DNA ligase, and Protein Molecular Weight Standards were purchased from Thermo Fisher Scientific (Shanghai, China). The TALON Metal Affinity Resin used for protein purification was obtained from TaKaRa. The DL-isocitrate and coenzymes (NAD^+^ and NADP^+^) were purchased from Sigma–Aldrich (Shanghai, China). All other biochemical reagents were obtained from Sangon Biotech and BBI (Shanghai, China).

### Recombinant Plasmid Construction

Based on the cDNA sequence of *IDH2* from the genome of *P. tricornutum* CCAP 1055/1 (NCBI accession number: CM000608.1), the codon-optimized open reading frame (ORF) encoding full-length *PtIDH2* was synthesized and cloned into the pET28b(+) vector by Generay Biotech (Shanghai, China). The truncated *PtIDH2* gene lacking the putative signal and transit peptide-encoding sequence (residues 1–58) was amplified and cloned into the expression vector to obtain pET-PtIDH2. The sequences of the primers used for gene amplification (PtIDH2-S and PtIDH2-As) are displayed in Table 1. The polymerase chain reaction (PCR) reaction program used for amplification was as follows: 95°C for 3 min, followed by 30 cycles of 95°C for 30 s, 58°C for 15 s, and 72°C for 35 s. The amplified products were digested with NdeI and XhoI and cloned into pET-28b(+). The gene was verified by DNA sequencing (General Biosystems, Chuzhou, China).

**TABLE 1:** The sequences of the primers used in this study.

Name^a^	Sequence (5'–3')^b^
PtIDH2-S	GGA​ATT​CCAT​ATGTTT​CGT​AGT​AGC​ACC​GTT​CTG​CT
PtIDH2-As	CCGCTC​GAGTTA​ATT​CAT​ATC​CGG​ACC​AAA​AGA​GGA​C
R664S-S	CGT​AAA​TCT​CCG​AGT***agc***GTG​GTT​AAT​CAG​ATT​G
R664S-As	CAA​TCT​GAT​TAA​CCA​C***gct***ACT​CGG​AGA​TTT​ACG
H604L-S	GCG​GTA​GCG​CCC​CGA​AA***ctg***GTT​CAG​CAG​TTT​GTT​AAA​G
H604L-As	CTT​TAA​CAA​ACT​GCT​GAA​C***cag***TTT​CGG​GGC​GCT​ACC​GC
K603T/H604L-S	CGG​TAG​CGC​CCC​G***acc​ctg***GTT​CAG​CAG​TTT​G
K603T/H604L-As	CAA​ACT​GCT​GAA​C***cag​ggt***CGG​GGC​GCT​ACC​G
R615D-S	GTT​AAA​GAA​GGT​CAC​TTA***gat***TGG​GAT​TCA​CTG​GGC​G
R615D-As	CGC​CCA​GTG​AAT​CCC​A***atc***TAA​GTG​ACC​TTC​TTT​AAC
V609L-S	CAT​GTT​CAG​CAG​TTT***ctg***AAA​GAA​GGT​CAC​TTA​C
V609L-As	GTA​AGT​GAC​CTT​CTT​T***cag***AAA​CTG​CTG​AAC​ATG
A718P-S	CAG​TGT​CAG​GGC​ACC***ccg***GTG​GAC​TTA​GGC​GGC
A718P-As	GCC​GCC​TAA​GTC​CAC***cgg***GGT​GCC​CTG​ACA​CTG
L742F-S	GAA​TCC​GAG​TCC​GAC​C***ttt***AAC​AAA​ATT​TTG​TCC​TC
L742F-As	GAG​GAC​AAA​ATT​TTG​TT***aaa***GGT​CGG​ACT​CGG​ATT​C
Tm-Mutants_As	CTG​CGA​TCC​CCG​GGA​AAA​CAG​CAT​TCC​AGG​TAT​TAG

a S and As, indicate the sense (S) and antisense (As) primers of the corresponding genes.

b Underlined bases indicate the restriction sites. CATATG, NdeI; CTCGAG, XhoI. Underlined bases in lowercase bold italic indicate the mutation site.

### Site-Directed Mutagenesis

Mutant forms of PtIDH2 (coenzyme specificity: H^604^L/R^615^D, H^604^L/R^615^D/R^664^S, and K^603^T/H^604^L/R^615^D/R^664^S; thermostability: V^609^L, A^718^P, and L^742^F) were generated by three rounds of site-directed mutagenesis (Ho et al., 1989). Primers for mutagenesis were designed according to structure-based amino acid sequence alignment (Table 1). PrimeSTAR Max DNA Polymerase was used along with one complementary pair of oligonucleotide primers for each mutation. The mutated genes were obtained through three successive PCR steps. Taking H^604^L as an example, the fragment upstream of the mutation site was amplifying using pET-PtIDH2 as the template using the primer pair PtIDH2-S and H^604^L-As, while the fragment downstream of the mutation site was amplified using the primer pair PtIDH2-As and H^604^L-S. Then, the two products, which contained overlapping fragments, were purified and joined by fusion PCR using the following program: 95°C for 3 min, followed by five cycles of 95°C for 30 s, 68°C for 30 s, and 72°C for 25 s. Finally, the full-length mutated gene was amplified with the primer pair PtIDH2-S and PtIDH2-As. The final fused PCR products containing the mutated sites were digested with NdeI and XhoI and ligated into the pET-28b(+) expression vector to create the recombinant plasmid pET-H^604^L. The recombinant plasmids harboring the mutated genes were transformed into competent *E. coli* DH5α cells. All mutated genes were verified by DNA sequencing (General Biosystems).

### Recombinant Protein Overexpression and Purification

The validated recombinant vectors were introduced into competent *E. coli* Rosetta (DE3) cells and cultured for 16 h at 37°C in Luria–Bertani (LB) medium containing 30 μg/ml chloramphenicol and 30 μg/ml kanamycin. The bacteria were then inoculated into 200 ml of fresh LB medium containing the same antibiotics and grown at 37°C with shaking (225 rpm) until the optical density at 600 nm (OD_600nm_) of the culture had reached 0.4–0.6. At this time, isopropyl-1-thio-β-D-galactopyranoside (IPTG) was added to the culture to a final concentration of 0.4 mM and the incubation was continued for 20 h at 20°C. The cells were harvested by centrifugation at 5,500 × *g* for 5 min at 4°C, resuspended in lysis buffer (20 mM Tris-HCl, 300 mM NaCl, 10% glycerol, pH 7.5), and disrupted by sonication. Cell debris was removed by centrifugation at 12,000 × *g* for 20 min at 4°C. Finally, recombinant PtIDH2 and its mutated forms fused with a 6×His tag were purified with TALON Metal Affinity Resin using a previously described method (Huang et al., 2020). The purity and subunit molecular mass of the recombinant enzyme were analyzed by 12% SDS–PAGE. The protein was stained with Coomassie Brilliant Blue R-250.

### Size-Exclusion Chromatography

The molecular mass of the recombinant PtIDH2 was estimated by size-exclusion chromatography (SEC) on the ÄKTA pure protein purification system equipped with a Superdex 200 (10/300) Increase column (GE Healthcare Life Sciences, Pittsburgh, PA, United States ). The columns were calibrated using the Gel Filtration HMW Calibration Kit (GE Healthcare) as described elsewhere (Huang et al., 2020). Five protein standards were used to calibrate the gels: ovalbumin (44 kDa), conalbumin (75 kDa), aldolase (158 kDa), ferritin (440 kDa), and thyroglobulin (669 kDa). The columns were equilibrated with two different buffers (20 mM Tris-HCl, 10% glycerol, pH 7.5, and either 300 mM or 1,000 mM NaCl). All protein samples were centrifuged at 12,000 × *g* for 20 min at 4°C. The protein was eluted at a flow rate of 0.5 ml/min and monitored at 280 nm.

SEC with multiangle light scattering (SEC–MALS) experiments were also performed using the ÄKTA pure protein purification system (GE Healthcare) coupled to a Wyatt DAWN HELEOS-II MALS instrument and a Wyatt Optilab rEX differential refractometer (Wyatt Technology, Santa Barbara, CA, United States ). Chromatographic separation was performed on a Superdex 200 (10/300) Increase column with a 100-μl sample loop at a flow rate of 0.5 ml/min in equilibration buffer (20 mM Tris-HCl, 10% glycerol, 300 mM NaCl, pH 7.5). The outputs were analyzed by ASTRAV 7.0 software (Wyatt Technology).

### Circular Dichroism Spectroscopy

Circular dichroism (CD) spectra were obtained with a Jasco model J-810 spectropolarimeter. Purified PtIDH2 and mutant protein samples were prepared in a CD buffer (20 mM NaH_2_PO_4_, 75 mM Na_2_SO_4_, and 10% glycerol, pH 7.5) to a final concentration of 0.2 mg/ml. The effects of temperature on the stability of PtIDH2 and mutants were determined by incubating sample at 20, 25, 30 and 35°C for 20 min, respectively. Subsequently, a microcuvette containing 200 μl of the protein sample was transferred into the instrument and scanned at the wavelength range of 190–280 nm. For each protein sample, the ellipticity (θ) was obtained by performing an average of three scans. The mean residue ellipticity ([θ], degcm^2^·dmole^−1^) was calculated as previously described (Huang et al., 2020). Protein secondary structure was estimated as described by Raussens et al. (2003).

### Enzyme Assays and Kinetic Characterization

The recombinant PtIDH2 was assayed by using an improved method described in Stokke et al. (2007). Enzyme assays for purified PtIDH2 and its mutated forms were carried out at 25°C in a 1-ml standard reaction mixture containing 50 mM Tris-HCl (pH 8.0), 1.0 mM DL-isocitrate, 2.0 mM MgCl_2_ or MnCl_2_, and 0.5 mM NADP^+^ or 5.0 mM NAD^+^. The reduction of NAD(P)^+^ (ε_340_ = 6.22 mM^−1^ cm^−1^) was monitored at 340 nm on a Carry 300 UV-Vis spectrophotometer (Agilent, Santa Clara, CA, United States ). One unit of enzyme activity (U or U/mg) refers to 1 μM NAD(P)H formed per minute. The protein concentrations were detected using a Bio-Rad Quick Start Bradford Protein Assay kit (Bio-Rad, Hercules, CA, United States ). All data are derived from at least three parallel tests.

The kinetic parameters of PtIDH2 were determined by measuring its enzyme activity at variable concentrations of DL-isocitrate and NAD(P)^+^. To the measurement of the apparent *K*_m_ for NADP^+^, the substrate—isocitrate concentration was kept fixed at 1.0 mM while the cofactor concentration (0.01–0.5 mM) varied. Similarly, to the measurement of the apparent *K*_m_ for isocitrate, the cofactor—NADP^+^ concentration was kept fixed at 0.5 mM whilst the isocitrate concentration (0.01–0.5 mM) varied. The *K*_m_ and *k*_cat_ values for the substrate and coenzyme were calculated by nonlinear fitting with GraphPad Prism 7.0 (GraphPad Software, San Diego, CA, United States ). All kinetic parameters represent at least three independent experiments.

The effects of pH on the activity of PtIDH2 were measured in 50 mM Tris-HCl buffer (25°C) between pH 6.8 and pH 9.0. The optimal temperature for the activity of PtIDH2 was determined in 50 mM Tris-HCl (pH 8.0) at a temperature range of 20–50°C. To investigate the thermostability of PtIDH2, aliquots of purified protein samples were incubated at 15–35°C for 20 min. Then, the residual enzyme activity was evaluated using standard assay reaction mixtures. The effects of different metal ions on recombinant PtIDH2 activity were also estimated using the previously described method (Wang et al., 2015). The activity of PtIDH2 was measured under an assay reaction mixture (25°C, 50 mM Tris, pH 8.0, 1.0 mM DL-isocitrate, and 0.5 mM NADP^+^) containing nine different metal ions (Na^+^, K^+^, Li^+^, Mn^2+^, Mg^2+^, Ca^2+^, Co^2+^, Cu^2+^, and Ni^2+^) at a 2.0 mM final concentration, respectively. The effects of various ions combinations on enzymatic activities were measured under the same assay reaction mixture in presence of either 2.0 mM MgCl_2_ or MnCl_2_. All values were normalized to the highest activity of PtIDH2 in each group. All data for enzyme activity were tested in at least three independent experiments.

## Results

### Phylogenetic Analysis and Sequence Alignment

The full-length *PtIDH2* gene comprises two introns and two exons, with a 2,433-bp ORF encoding a putative polypeptide of 811 amino acids (pre-PtIDH2), which is typical for members of the type III IDH subfamily. To the best of our knowledge, PtIDH2 is the first type III IDH identified in eukaryotes. Additionally, we found that relatively few eukaryotic genomes encode this type of IDH, most of which belong to the marine stramenopile lineage, including heterokonts, haptophytes, and cryptophytes. Multiple amino acid sequence alignment indicated that PtIDH2 shares the highest homology with marine bacterial monomeric NADP-IDHs, such as *Psychromonas marina* IDH (56%), *Colwellia maris* IDH (54%), and *Colwellia psychrerythraea* IDH (54%). Numerous studies have indicated that both *P. tricornutum* and the aforementioned marine bacteria have potential overlapping habitats in marine and polar ecosystems, suggesting that these novel eukaryotic type III IDHs may have originated from marine prokaryotes *via* widespread horizontal gene transfer.

**FIGURE 1:**
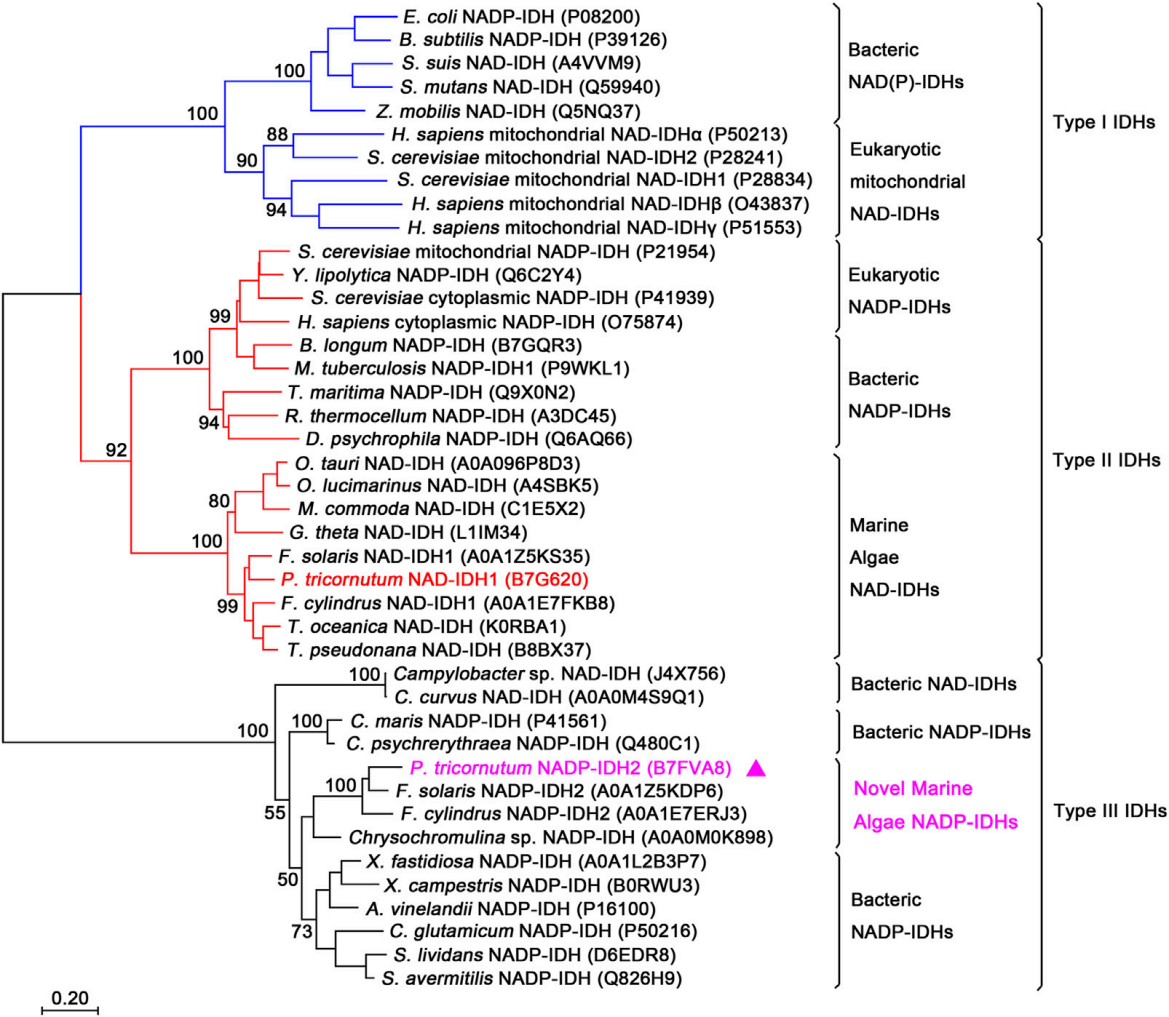
Phylogenetic analysis of the isocitrate dehydrogenase (IDH) protein family. The analysis included 42 IDH sequences. A neighbor-joining tree with 1,000 bootstrap replicates was constructed using MEGA7. The UniProt entry IDs of all the IDHs are noted in parentheses. PtIDH2 is represented by triangles.

The type III IDH subfamily was recently redefined through the addition of a novel monomeric NAD-IDH subgroup, which highly likely comprises the ancestral proteins of this subfamily (Wang et al., 2015; Wang et al., 2019). To determine the evolutionary relationship between PtIDH2 and other IDH protein family members, a phylogenetic tree was reconstructed in MEGA7 using 42 IDH sequences. The result clearly indicated that PtIDH2 and other novel marine algae (eukaryotic) type III IDHs fell into one cluster that was closest to the clade of monomeric NADP-IDHs found in marine bacteria (Figure 1). The other type III NAD(P)-IDHs from prokaryotes were divided into individual clades. The phylogenetic analysis suggested that PtIDH2 is a novel member of the NADP^+^-specific type III IDH subfamily.

**FIGURE 2:**
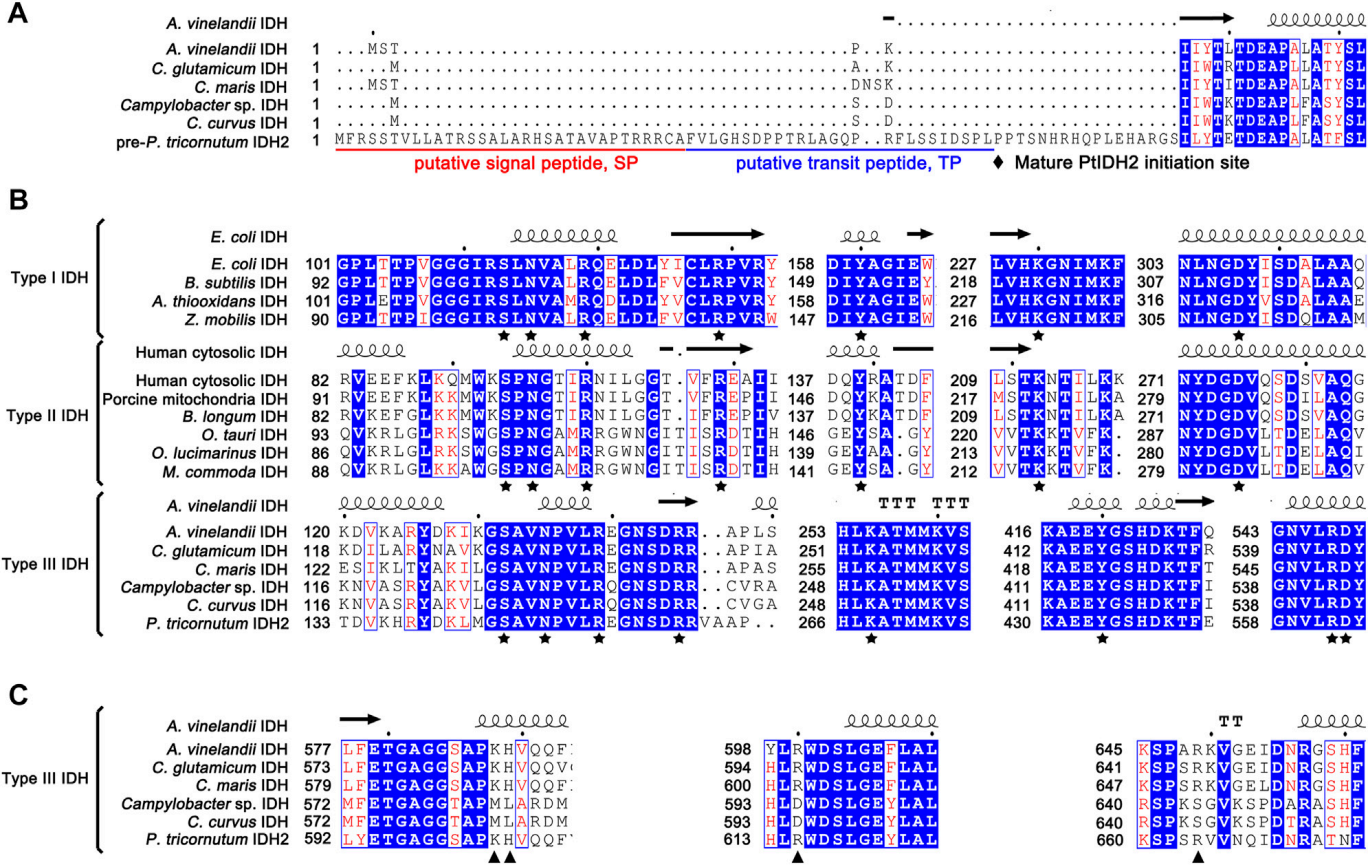
Structure-based protein sequence alignment of PtIDH2 with other isocitrate dehydrogenases (IDHs). **(A)** Comparison of the N-terminal amino acid sequence of PtIDH2 with those of other bacterial monomeric IDHs. The putative signal peptide and target peptide sequences are underlined in red and blue and possible cleavage sites are indicated by arrows. **(B)** The conserved substrate-binding amino acid residues of the IDH protein family are indicated by pentagrams. **(C)** The putative conserved residues implicated in PtIDH2 coenzyme binding are compared with those of other monomeric NAD(P)-IDHs. The residues that directly or indirectly interact with the 2′-phosphate of NADP^+^ are indicated by triangles.

Additionally, subcellular localization prediction indicated that PtIDH2 was located in the plastid and was predicted to contain a putative N-terminal 58-amino acid signal and transit peptide (Figure 2A). To identify potential functional sites in PtIDH2, a structure-based multiple amino acid sequence alignment was carried out (Figure 2). IDHs are single-substrate, metal-dependent enzymes. All the residues involved in substrate- and metal ion-binding, including Ser, Asn, Arg, Tyr, Lys, and Asp (Figure 2B), are completely conserved in type I, II, and III IDH subfamily members. However, the putative critical residues of PtIDH2 involved in coenzyme binding differ significantly from those of monomeric NAD-IDHs (Figure 2C). It has been shown that monomeric NAD-IDHs, such as *Campylobacter* sp. IDH (CaIDH) and *Campylobacter curvus* IDH (CcIDH) (Wang et al., 2015; Wang et al., 2019), primarily use polar, uncharged amino acids (Met583, Leu584, Asp595, and Ser644 for CaIDH) to bind the coenzyme NAD^+^. In contrast, the residues that bind the coenzyme NADP^+^ in type III NADP-IDHs are positively charged (His and Arg). The crystal structure of monomeric NADP-IDH from *A. vinelandii* revealed that the side chains of His589 and Arg600 form hydrogen bonds with the 2′-phosphomonoester of NADP^+^, and play a determinant role in coenzyme specificity (Imabayashi et al., 2006). Additionally, three key residues of PtIDH2 involved in coenzyme specificity (His604, Arg615, and Arg664) were found to be identical to the corresponding residues in AvIDH (Supplementary Figure S1), implying that PtIDH2 might use NADP^+^ as a coenzyme.

### Overexpression and Purification

**FIGURE 3:**
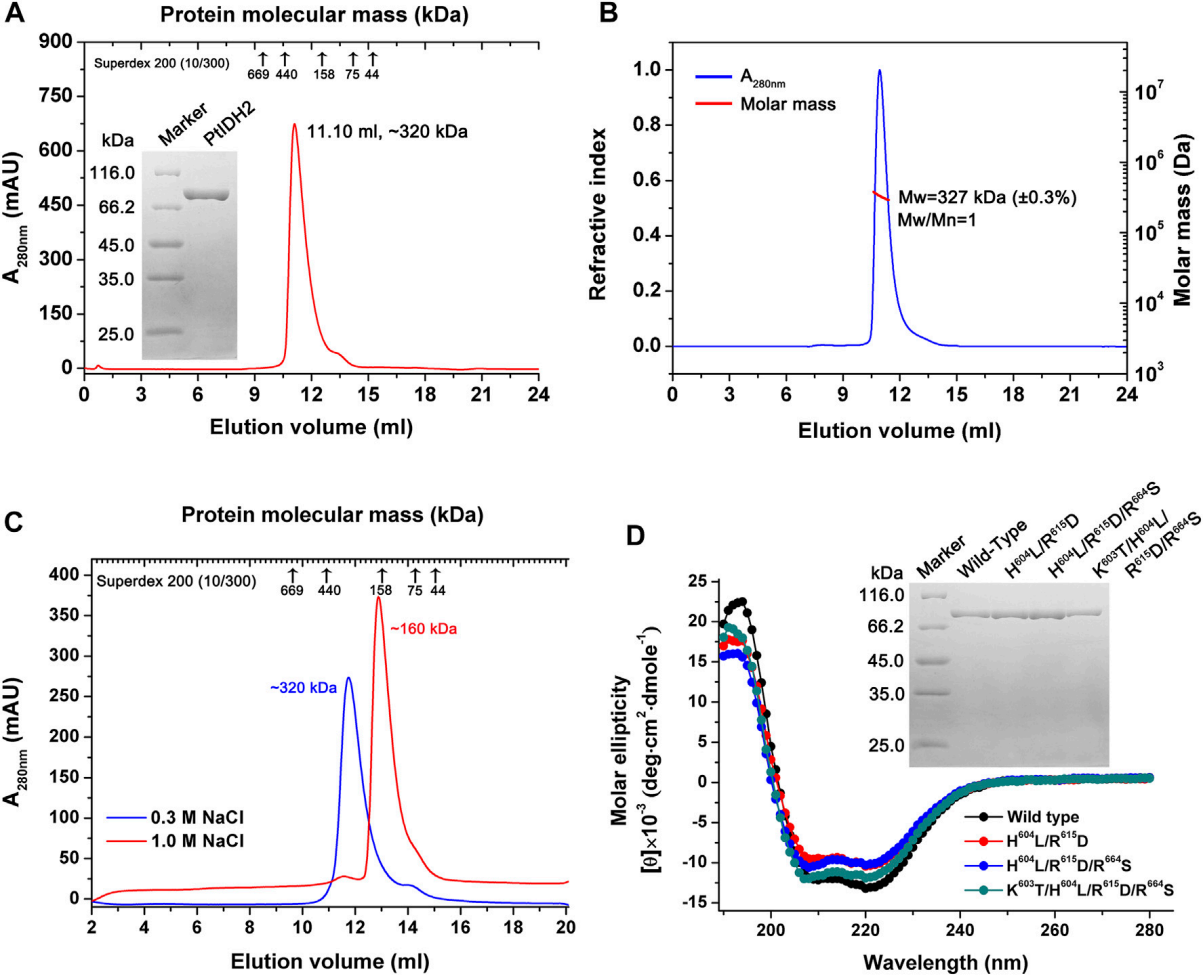
Overexpression and purification of PtIDH2 and its mutant forms. **(A)** Overexpression and purification of wild-type PtIDH2. Size-exclusion chromatography (SEC) elution profile of PtIDH2 obtained using a Superdex 200 (10/300) column at a flow rate of 0.5 ml/min. The left inset panel shows the protein purity detection by 12% SDS–PAGE. **(B)** SEC with multiangle light scattering (SEC–MALS) analysis of wild-type PtIDH2. The molar mass and refractive index were plotted *vs.* the elution volume from a Superdex 200 (10/300) column with a flow rate of 0.5 ml/min. **(C)** SEC elution profiles of PtIDH2 performed with Superdex 200 (10/300) column. The column was equilibrated with a buffer (20 mM Tris-HCl, 10% Glycerol, pH 7.5) containing 0.3 M or 1.0 M NaCl, respectively. PtIDH2 samples were also incubated in that buffer before chromatography. **(D)** Circular dichroism spectra of wild-type PtIDH2 and three mutants. The right inset panel shows the wild-type PtIDH2 and three mutants purity detection by 12% SDS–PAGE.

The mature PtIDH2 minus the signal and transit peptide was heterologously expressed in *E. coli* cells and purified using Co^2+^ affinity chromatography. The SDS–PAGE analysis showed that recombinant 6×His-tagged PtIDH2 had a molecular mass of approximately 80 kDa, which was consistent with its theoretical molecular mass (∼82 kDa). The length of the polypeptide chain of mature PtIDH2 (753 amino acids) and phylogenetic analysis suggested that PtIDH2 was a typical monomeric protein. However, SEC indicated that PtIDH2 eluted as a single symmetric peak with a molecular mass of approximately 320 kDa, demonstrating that PtIDH2 formed a homotetramer in solution (Figure 3A). SEC–MALS was used to further determine the absolute molecular mass of PtIDH2 in solution with greater accuracy. The result showed that PtIDH2 eluted as a prominent monodisperse peak with an average molecular mass of 327 kDa, which was nearly four-fold that of the subunit molecular weight, suggesting that PtIDH2 indeed adopts a homotetrameric conformation in solution (Figure 3B).

To evaluate whether the oligomeric state of recombinant PtIDH2 is influenced by ionic strength, the natural molecular mass of PtIDH2 was estimated under both low (0.3 M NaCl) and high (1.0 M NaCl) salting strength conditions. Surprisingly, the elution profiles of PtIDH2 showed a single peak corresponding to a homotetramer (∼320 kDa) under low salt condition, which was dissociated into a homodimer (∼160 kDa) at high salt condition, but not into a monomer (Figure 3C). These results provided a strong evidence that the natural homotetrameric structure of recombinant PtIDH2 was affected by ionic strength. It demonstrated that PtIDH2 can exist in higher oligomeric forms, and homodimer represented the most stable form that could not be further dissociated in different buffer conditions. The above result was very similar to the NADP-IDH2 from pathogenic bacteria *Mycobacterium tuberculosis* (Banerjee, et al., 2005). However, the physiological relevance of different oligomers is currently unknown.

### Analysis of PtIDH2 Kinetics

The recombinant PtIDH2 exhibited a complete preference for NADP^+^ and displayed no detectable NAD^+^-associated catalytic activity, in line with the above bioinformatic analysis. The specific activity with NADP^+^ was determined to be 82.02 ± 5.04 and 18.21 ± 2.65 U/mg in the presence of Mn^2+^ and Mg^2+^, respectively. The apparent *K*_m_ values of recombinant PtIDH2 for isocitrate were 42.69 ± 8.2 μM with Mn^2+^ and 14.24 ± 1.01 μM with Mg^2+^ when using NADP^+^ as the coenzyme, and the *k*_cat_/*K*_m_ values for isocitrate were respectively 2.90 and 1.80 μM^−1^ s^−1^ with Mn^2+^ and Mg^2+^. Additionally, the *K*_m_ values of PtIDH2 for NADP^+^ were 37.37 ± 4.02 μM with Mn^2+^ and 18.37 ± 2.94 μM with Mg^2+^, and the corresponding catalytic efficiency (*k*_cat_/*K*_m_) values were 3.22 and 2.36 μM^−1^ s^−1^ (Supplementary Figure S2). These *k*_cat_/*K*_m_ values were similar to those for the bacterial monomeric NADP-IDHs from *Xanthomonas campestris* (6.0 μM^−1^ s^−1^ with Mn^2+^) and *Streptomyces avermitilis* (11.7 μM^−1^ s^−1^ with Mn^2+^) (Wang et al., 2011; Lv et al., 2016), but higher than those for the homodimeric NADP-IDH (contains two monomeric IDH-like subunits) from *A. baumannii* (0.39 μM^−1^ s^−1^ with Mn^2+^) (Wang et al., 2018) and lower than those for the monomeric IDHs from *A. vinelandii* (15.0 μM^−1^ s^−1^ with Mn^2+^) and *Xylella fastidiosa* (96.5 μM^−1^ s^−1^ with Mn^2+^) (Watanabe et al., 2005; Lv et al., 2018).

Through amino acid sequence alignment and homology modeling structure comparison with other type III NAD^+^ or NADP^+^-specific IDHs, four residues (Lys603, His604, Arg615, and Arg664) were identified as potentially critical NADP^+^-binding sites in PtIDH2 (Supplementary Figure S1). To further examine the determinants for PtIDH2 coenzyme specificity, three mutant PtIDH2 forms (H^604^L/R^615^D, H^604^L/R^615^D/R^664^S, and K^603^T/H^604^L/R^615^D/R^664^S) were generated by site-directed mutagenesis (Figure 3D) based on CaIDH (monomeric NAD-IDH). The CD spectra of the mutated proteins were similar to those of wild-type PtIDH2, demonstrating that mutations did not induce conformational changes in the secondary structure of the enzyme (Figure 3D).

The kinetic parameters of PtIDH2 and those of its mutant forms are listed in Table 2. The H^604^L/R^615^D double mutant displayed significantly decreased affinity (1 /*K*_m_, approximately 1,275-fold) and catalytic efficiency (*k*_cat_/*K*_m_, approximately 6,440-fold) toward the NADP^+^ cofactor when compared with those for wild-type PtIDH2. Meanwhile, the H^604^L/R^615^D mutant also showed a slight catalytic activity for NAD^+^, suggesting that His604 and Arg615 were major determinants of coenzyme specificity in PtIDH2. A third point mutation (R^664^S) was subsequently introduced, generating the H^604^L/R^615^D/R^664^S triple mutant. Mutations at these three sites led to a 72-fold increase in the *K*_m_ value and an 8,050-fold decrease in catalytic efficiency for NADP^+^ with respect to wild-type PtIDH2. Furthermore, the affinity and catalytic efficiency of the H^604^L/R^615^D/R^664^S triple mutant toward NAD^+^ were further increased by approximately 1.7-fold and 2.0-fold, respectively. To obtain a better conversion effect, the adjacent Lys603 was substituted with Thr, which generated the K^603^T/H^604^L/R^615^D/R^664^S PtIDH2 quadruple mutant. Unexpectedly, this mutant exhibited no detectable activity in a NADP^+^-linked catalytic reaction, while its affinity and catalytic efficiency toward NAD^+^ were also very poor when compared with those of the H^604^L/R^615^D/R^664^S triple mutant. These results suggested that the adjacent Thr residue does not contribute to NAD^+^-specificity. The PtIDH2 H^604^L/R^615^D/R^664^S mutant displayed an approximately 3.25-fold preference for NAD^+^ over NADP^+^ [(*k*_cat_/*K*_m_^NAD^)/(*k*_cat_/*K*_m_^NADP^)], which indicated that the coenzyme specificity of PtIDH2 had been completely converted from NADP^+^ to NAD^+^ by the triple mutation.

**TABLE 2:** Kinetic parameters of wild-type PtIDH2 and those of its mutant forms.

Enzyme	NADP^+^			NAD^+^			
	*K*_m_	*k*_cat_	*k*_cat_/*K*_m_		*K*_m_	*k*_cat_	*k*_cat_/*K*_m_
	(μM)	(s^−1^)	(μM^−1^ s^−1^)		(μM)	(s^−1^)	(μM^−1^ s^−1^)
PtIDH2	37.37 ± 4.02	118.89 ± 11	3.22		—	—	—
H^604^L/R^615^D	47,650 ± 8,344	22.13 ± 5.5	0.0005		10,271 ± 1,448	7.22 ± 0.60	0.0007
H^604^L/R^615^D/R^664^S	2,704 ± 142.8	1.01 ± 0.07	0.0004		5,825 ± 307	7.67 ± 1.04	0.0013
K^603^T/H^604^L/R^615^D/R^664^S	—	—	—		11,479 ± 299	2.25 ± 0.18	0.0002

“-“, No detectable activity. Data are presented as means ± SD of at least three independent measurements.

### The Effects of pH, Temperature, and Metal Ions

**FIGURE 4:**
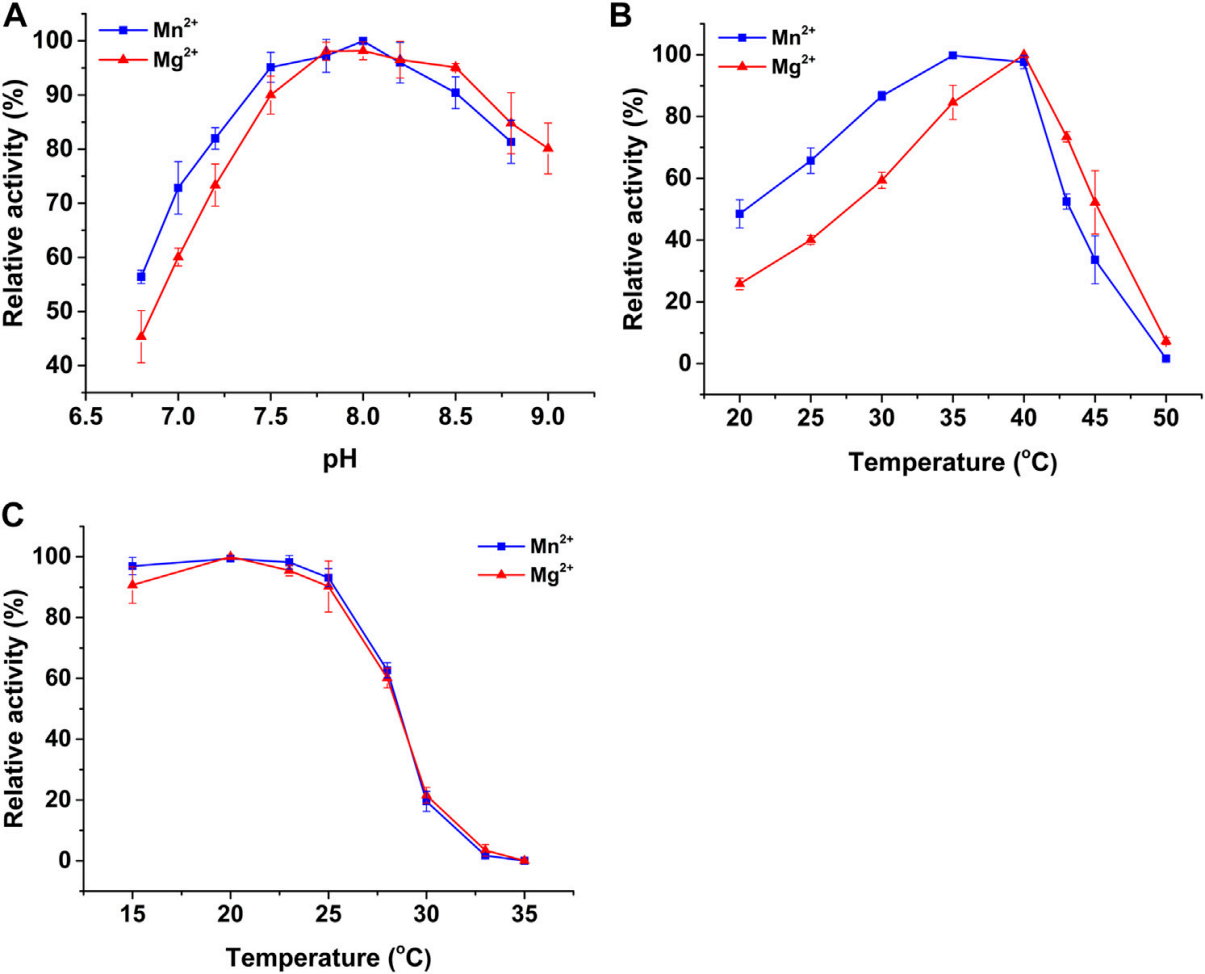
The effects of pH and temperature on the NADP^+^-linked activity of PtIDH2 in the presence of Mn^2+^ (blue squares) and Mg^2+^ (red triangles). **(A)** The effects of pH on the activity of PtIDH2. **(B)** The effects of temperature on the activity of PtIDH2. **(C)** Heat-inactivation profiles of PtIDH2. All values are displayed as means ± SD of at least three independent measurements.

The effects of pH and temperature on the activity of recombinant PtIDH2 were determined in a NADP^+^-associated catalytic reaction. Recombinant PtIDH2 retained more than 60% activity across a wide pH range (7.5–9.0) (Figure 4A). The optimum pH value for PtIDH2 was approximately 8.0 regardless of whether Mn^2+^ or Mg^2+^ was used, similar to that observed for the bacterial monomeric NADP-IDHs from *Methanococcoides methylutens* (pH 8.2 with Mn^2+^ and 8.5 with Mg^2+^) and *Streptomyces lividans* (pH 8.5 with Mn^2+^) (Zhang et al., 2009; Wang et al., 2020), but lower than that for *Streptomyces avermitilis* IDH (pH 9.4 with Mn^2+^) and *C. glutamicum* IDH (pH 9.0 with Mg^2+^) (Chen and Yang, 2000; Wang et al., 2011) and higher than that for *X. fastidiosa* IDH (pH 7.75 with Mn^2+^) (Lv et al., 2018).

The optimum temperature for PtIDH2 catalysis was 35 and 40 °C in the presence of Mn^2+^ and Mg^2+^, respectively (Figure 4B). The enzymatic activity of PtIDH2 decreased rapidly at temperatures above 40 °C and was almost entirely lost at 50°C. These values were very close to those for IDHs from other marine bacteria, namely, *Congregibacter litoralis* (35°C with Mn^2+^ and Mg^2+^), *P. marina* (35 °C with Mn^2+^), and *C. psychrerythraea* (30°C with Mn^2+^) (Wu et al., 2015; Suzuki and Takada, 2016; Hirota et al., 2017), but lower those for *A. vinelandii* IDH (55°C with Mn^2+^) and *P. psychrophila* IDH (50°C with Mn^2+^) (Matsuo et al., 2010; Tsubouchi and Takada, 2019). The heat-inactivation profiles suggested that PtIDH2 was stable under 25°C, but its stability decreased rapidly with incubation at higher temperatures. PtIDH2 retained only 60% residual catalytic activity when incubated at 28°C for 20 min (Figure 4C).

IDHs are dependent on metal ions as activators to initiate catalytic reactions. Here, we examined the effects of three monovalent and six divalent metal ions on the activity of recombinant PtIDH2. The results showed that the activity of PtIDH2 was entirely dependent on the presence of divalent metal cations (Table 3), with Mn^2+^ being the most effective activator for PtIDH2, followed by Mg^2+^. Compared with Mn^2+^, the activity of PtIDH2 was reduced by 70% in the presence of Mg^2+^. None of the other metal cations were capable of activating PtIDH2. Meanwhile, Ca^2+^, Cu^2+^, and Ni^2+^ strongly inhibited the activity of PtIDH2 in the presence of Mn^2+^ and Mg^2+^. The monovalent metal ions Na^+^, K^+^, and Li^+^ had little effect on the activity of PtIDH2. Interestingly, Mn^2+^ appears to be the optimal activator for most monomeric IDHs, including *Streptomyces lividans* IDH, as well as for type II homodimeric IDHs such as *P. tricornutum* IDH1 and *Bifidobacterium longum* IDH (Zhang et al., 2009; Huang et al., 2016; Huang et al., 2020).

**TABLE 3:** The effects of different metal ions on the activity of PtIDH2.

Metal ion	Relative activity (%)	Metal ion(s)	Relative activity (%)	Metal ion(s)	Relative activity (%)
None	2.21 ± 0.50	—	—	—	—
Mn^2+^	100	Mn^2+^	100	Mg^2+^	100
Mg^2+^	29.92 ± 1.37	Mn^2+^+Mg^2+^	100.90 ± 2.17	Mg^2+^+Mn^2+^	337.82 ± 20.38
Ca^2+^	0.96 ± 1.10	Mn^2+^+Ca^2+^	33.88 ± 0.65	Mg^2+^+Ca^2+^	11.20 ± 1.19
Co^2+^	13.16 ± 0.72	Mn^2+^+Co^2+^	96.42 ± 0.94	Mg^2+^+Co^2+^	91.55 ± 1.48
Cu^2+^	1.96 ± 0.17	Mn^2+^+Cu^2+^	86.75 ± 1.81	Mg^2+^+Cu^2+^	83.02 ± 3.55
Ni^2+^	2.03 ± 0.37	Mn^2+^+Ni^2+^	92.95 ± 5.52	Mg^2+^+Ni^2+^	64.37 ± 1.71
Na^+^	2.07 ± 0.39	Mn^2+^+Na^+^	101.23 ± 4.41	Mg^2+^+Na^+^	99.52 ± 0.84
Li^+^	2.53 ± 0.96	Mn^2+^+Li^+^	101.06 ± 2.21	Mg^2+^+Li^+^	105.95 ± 6.56
K^+^	2.63 ± 0.37	Mn^2+^+K^+^	96.32 ± 2.27	Mg^2+^+K^+^	99.09 ± 5.56

The relative activity was assessed in a standard reaction mixture containing 2 mM of the indicated metal ion(s). Data are expressed as means ± SD of at least three independent measurements.

### Thermal Properties of Wild–Type and Mutated PtIDH2

**FIGURE 5:**
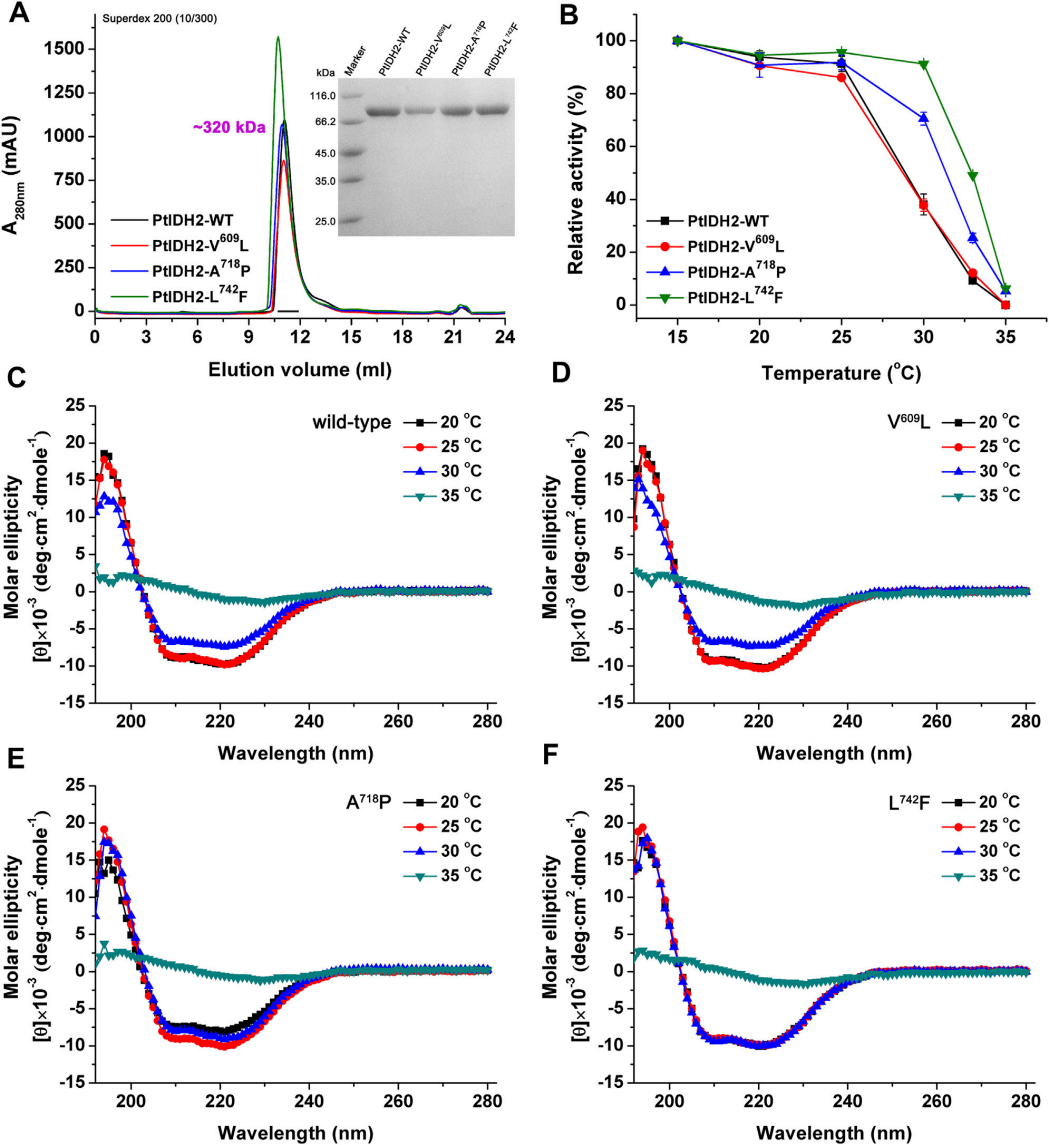
Overexpression and purification of mutated PtIDH2. **(A)** Overexpression, purification, and determination of the oligomeric state of mutated PtIDH2. Size-exclusion chromatography elution profiles of mutant forms of PtIDH2 obtained using a Superdex 200 (10/300) column at a flow rate of 0.5 ml/min. The right inset panel shows the protein purity detection by 12% SDS–PAGE. **(B)** Heat-inactivation profiles of the wild-type and mutated forms of PtIDH2. All values are displayed as means ± SD of at least three independent measurements. **(C)** CD spectra of four states of wild-type PtIDH2. The individual enzymes were incubated for 20 min at 20, 25, 30, and 35°C, respectively. **(D)** CD spectra of four states of V^609^L. The individual enzymes were incubated for 20 min at 20, 25, 30 and 35°C, respectively. **(E)** CD spectra of four states of A^718^P. The individual enzymes were incubated for 20 min at 20, 25, 30 and 35°C, respectively. **(F)** CD spectra of four states of L^742^F. The individual enzymes were incubated for 20 min at 20, 25, 30 and 35°C, respectively.

Although PtIDH2 and AvIDH are both members of the type III IDH subfamily and have highly homologous amino acid sequences (53%), their thermal properties differ significantly. As mentioned above, PtIDH2 shows the highest activity at 35°C and loses over 80% of its activity when incubated at 30°C for 20 min, demonstrating that it is a cold-adapted enzyme. In contrast, the optimum temperature for the activity of monomeric AvIDH is 55°C, and this activity is maintained even after 10 min of incubation at 40°C, suggesting that AvIDH is a mesophilic enzyme (Tsubouchi and Takada, 2019). Generally, cold-adapted enzymes are markedly thermolabile and show high catalytic activity at low temperatures, the opposite of that observed for mesophilic and hyperthermophilic enzymes. Several critical residues located at the C-terminus (known as Region III) of monomeric IDHs are known to directly determine their thermostability (Yasutake et al., 2003; Watanabe et al., 2005). The alignment of PtIDH2 with some mesophilic and cold-adapted monomeric NADP-IDHs suggested that three residues (Val609, Ala718, and Leu742) may be critical for the thermostability of PtIDH2, corresponding to Leu594, Pro709, and Phe733 of AvIDH (Supplementary Figure S3). To verify the determinants of PtIDH2 thermostability, three substitutional mutations of these residues were generated by site-directed mutagenesis. SEC analysis revealed that the mutants retained their native homotetrameric structure (Figure 5A), suggesting that the substitutional mutations did not change the conformation of the enzyme.

Enzyme assays showed that the specific activities of the V^609^L, A^718^P, and L^742^F PtIDH2 mutants were 76.42 ± 1.62, 72.25 ± 3.76, and 83.19 ± 1.11 U/mg, respectively, in the presence of Mn^2+^ and NADP^+^ at 25°C, values that were comparable with those of wild-type PtIDH2 (82.02 ± 5.04 U/mg). These data demonstrated that Val609, Ala718, and Leu742 are distant from the active catalytic center of the enzyme and do not directly affect the interaction of the enzyme with its substrate and coenzyme when the individual amino acids are substituted. We next investigated the thermostability of wild-type PtIDH2 and that of its mutant forms (Figure 5B). Following incubation at temperatures of up to 25°C, the wild-type enzyme retained most of its activity; however, its residual activity rapidly decreased by incubation at temperatures above 25°C, and was lost after incubation for 20 min at 35°C. The V^609^L mutant had a similar heat-inactivation profile to that of the wild-type enzyme, indicating that the Leu at position 609 is not involved in the thermostability of PtIDH2. Nevertheless, the other two mutant enzymes (A^718^P and L^742^F) were more thermostable than wild-type PtIDH2. Analysis of their heat-inactivation profiles showed that the rate of descent of the activity of the A^718^P mutant was significantly slower than that of the wild-type enzyme with incubation at temperatures above 25°C. Approximately 71% of the activity of this mutant was retained with incubation at 30°C, contrasting with the 38% residual activity of wild-type PtIDH2 after incubation at the same temperature. These data indicated that the L^742^F mutant form of PtIDH2 was the most thermostable of the three mutants. The residual activity of this mutant after incubation at 30°C, 33°C, and 35°C was 91, 50, and 6%, respectively. Moreover, CD spectroscopy was used to evaluate the effects of temperature on the secondary-structure elements contents of PtIDH2 and mutants. PtIDH2 wild-type and V^609^L seemed to be equally sensitive to temperature above 30°C, and the CD spectra of them after incubated to 35°C were significantly different from the spectrum of the natural enzyme (Figures 5C,D). A^718^P appeared to be more stable after incubated at 30 °C, but it was also observed degeneration at temperature of 35°C (Figure 5E). Almost no change in the CD spectra characteristics of L^742^F was observed after incubation for 20 min at 30 °C (Figure 5F), indicating that L^742^F is more thermostable than other three enzyme which we evaluated. Obviously, there was a positive correlation between the heat-inactivation profiles and CD spectra. These results demonstrated that Ala718 and Leu742 are the major determinants of the cold-adapted activity of PtIDH2, and substitutional mutations of these critical residues located at the C-terminus result in an additive increase in its thermostability with respect to catalytic activity.

## Discussion

Based on phylogeny, the IDH protein family has been classified into three subfamilies, namely, types I, II, and III. Additionally, two types of IDHs—monomeric and homodimeric NADP-IDHs—can be distinguished in the type III subfamily based on coenzyme specificity and oligomeric state (Wang et al., 2015; Wang et al., 2018). All type III IDHs reported to date are derived from prokaryotes, including both bacteria and archaea (Wang et al., 2020). In this study, a novel type III homotetrameric NADP-IDH from the marine alga *P. tricornutum*, a eukaryote, was characterized in detail for the first time. BLAST analysis revealed that the distribution of type III IDHs in eukaryotes is restricted, with only a relatively few strains of marine unicellular algae encoding this type of IDH (Supplementary Table S1). Accordingly, we reconstructed the phylogenetic tree of the IDH protein family and redefined the type III subfamily by adding a new group of eukaryotic homotetrameric NADP-IDHs (Figure 1) that are composed of four monomeric IDH-like subunits. This finding further expands the current phylogeny and classification of the IDH protein family.

Kinetic studies revealed that PtIDH2 is a completely NADP^+^-specific IDH. Moreover, the *K*_m_ of PtIDH2 for NADP^+^ (37.4 μM) was slightly higher than those of typical monomeric NADP-IDHs such as *X. campestris* IDH (17.5 μM) (Lv et al., 2016), *A. vinelandii* IDH (5.8 μM) (Tsubouchi and Takada, 2019), *S. avermitilis* IDH (5.0 μM) (Wang et al., 2011), *C. glutamicum IDH* (4.0 μM) (Chen and Yang, 2000), and *X. fastidiosa* IDH (1.0 μM) (Lv et al., 2018), but lower than that of the homodimeric IDH from *A. baumannii* (94.0 μM) (Wang et al., 2018) (Supplementary Table S2). This indicates that the affinity of PtIDH2 for NADP^+^ is lower than that of monomeric NADP-IDHs, but higher than that of the homodimeric NADP-IDHs of the type III subfamily.

We have previously shown that NAD^+^ specificity is an ancestral IDH phenotype, while NADP^+^ dependency likely represents a subsequent bacterial adaptation to a barren environment (Zhu et al., 2005). The IDH protein family has evolved conserved amino acid residues as determinants of coenzyme binding, and the coenzyme specificity of IDHs is governed by the combined interactions of surrounding residues with NAD^+^ or NADP^+^. Crystallographic analysis of AvIDH revealed that this enzyme mainly uses positively charged, polar amino acids, including His589, Arg600, and Arg649, to bind the NADP^+^ cofactor *via* hydrogen bonds and strong ion-pair interactions (Imabayashi et al., 2006). In contrast, Leu584, Asp595, and Ser644 in CaIDH, which correspond, respectively, to the NADP^+^ binding residues in AvIDH, are polar uncharged amino acids. The side chains of Asp and Leu form hydrogen bonds with the 2′ and 3′-hydroxyl groups of NAD^+^, such that the enzyme has a high affinity for this coenzyme (Wang et al., 2015; Wang et al., 2019).

Amino acid sequence alignment and available structural information indicated that the His604, Arg615, and Arg664 residues in PtIDH2 were likely to be the putative NADP^+^ binding sites through directly interacting with the 2′-phosphate group of the coenzyme. Accordingly, we undertook a site-directed mutagenesis-based analysis of these potential critical sites, which led to the transformation of the coenzyme specificity of PtIDH2 from NADP^+^ to NAD^+^. Although the H^604^L/R^615^D double mutant enzyme displayed a substantial decrease in affinity and catalytic efficiency toward NADP^+^, it showed only an approximately 1.4-fold preference for NAD^+^ over NADP^+^, suggesting that the coenzyme specificity of PtIDH2 was not altered when two residues were substituted. To then improve the affinity and catalytic activity of PtIDH2 for NAD^+^, a third mutation—R^664^S—was introduced into the H^604^L/R^615^D mutant enzyme. This triple mutant showed an approximately 2-fold increase in affinity and catalytic activity for NAD^+^ when compared with that seen with the double mutant. Additionally, the preference of the H^604^L/R^615^D/R^664^S mutant for NAD^+^ was 3.25-fold that for NADP^+^, showing that the coenzyme specificity of PtIDH2 had converted from NADP^+^ to NAD^+^ through a rational engineering approach. Interestingly, even though the H^604^L/R^615^D/R^664^S mutant enzyme could utilize NAD^+^ as the coenzyme, the catalytic efficiency was fairly low. Similar results were obtained in other studies that sought to convert the coenzyme dependence of IDH from NADP^+^ to NAD^+^, such as for *M. methylutens* IDH (Wang et al., 2020), *X. fastidiosa* IDH (Lv et al., 2018), and *X. campestris* IDH (Lv et al., 2016) (Supplementary Table S2). These results indicated that additional amino acid residues are involved in the binding of NAD^+^ by monomeric IDHs, and further imply that the mechanisms involved in NAD^+^ catalysis are more complex than those involved in NADP^+^ catalysis in the type III IDH subfamily. These observations highlight the need to resolve the crystal structure of monomeric NAD-IDHs.

**FIGURE 6:**
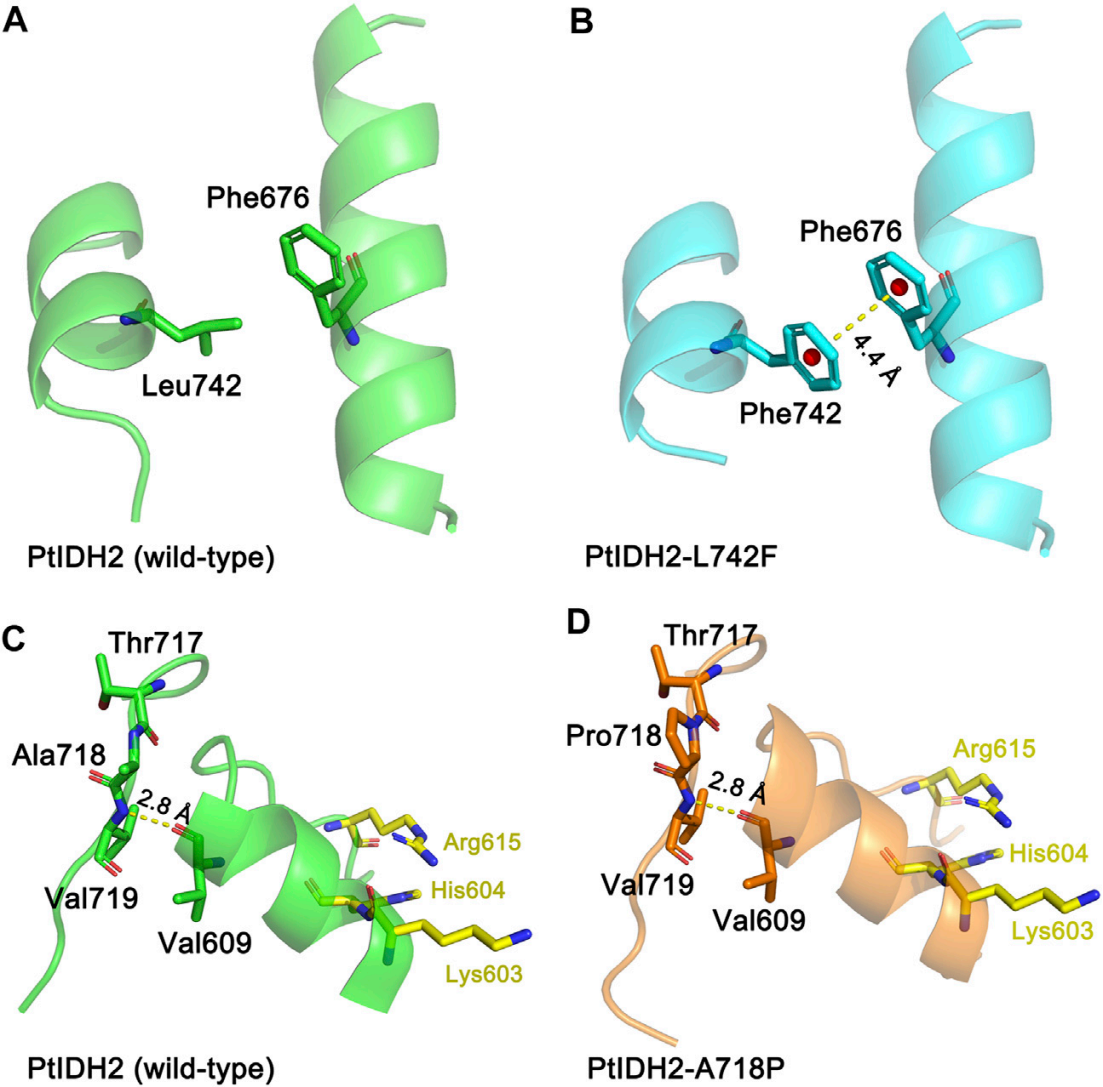
Molecular models of wild-type and mutated PtIDH2. **(A)** The locations of Leu742 and Phe 676 in wild-type PtIDH2. **(B)** The locations of Phe742 and Phe676 in the L^742^F mutant. The yellow dashed line shows an aromatic–aromatic interaction at a distance of 4.4 Å. **(C)** The locations of Thr717, Ala718, Val719, and Val609 in wild-type PtIDH2. **(D)** The locations of Thr717, Pro718, Val719, and Val609 in the A^718^P mutant. The yellow dashed line shows a hydrogen bond with a length of 2.8 Å. The PtIDH2 model and that of its mutants were built by the SWISS-MODEL web-server using the *A. vinelandii* isocitrate dehydrogenase (IDH) (PDB code: 1ITW) as a template.

Enzymatic characterization demonstrated that PtIDH2 has high catalytic activity at low temperatures, and that it is a thermolabile enzyme similar to that seen in the monomeric IDHs from marine psychrophilic bacteria, such as *C. maris*, *C. psychrerythraea*, and *P. marina* (Tsubouchi and Takada, 2019; Nagai and Takada, 2020). The results of this study demonstrated that substituting a few amino acid residues in the C-terminus, including Ala718 and Leu742 with Pro and Phe, respectively, induced marked changes in the thermostability of PtIDH2. The L^742^F mutant showed the highest thermostability of all the mutated PtIDH2 forms, indicating that Leu742 is involved in the catalytic activity-related thermostability of PtIDH2. In the structure model of the L^742^F mutant, the distance between the side chains of Phe742 and Phe676 was approximately 4.4 Å, which was very close. It is well known that an aromatic–aromatic interaction will be formed when the benzene ring centroids of aromatic side chains in two proteins are separated by a distance of between 4.5 Å and 7.0 Å. Therefore, an aromatic–aromatic interaction between Phe742 and Phe676 may have contributed to the structural stabilization of PtIDH2, thereby enhancing its thermostability (Figures 6A,B). The A^718^P mutation also slightly increased the thermostability of PtIDH2. The substitution of Ala718 by Pro may decrease the flexibility of the loop, contributing to an increase in the thermostability of the protein (Figures 6C,D). Similar results were obtained when the Ala and Leu residues of cold-adapted monomeric NADP-IDHs from marine psychrophilic bacteria were substituted, such as in *P. marina* (Hirota et al., 2017; Tsubouchi and Takada, 2019). This indicates that the residues that are critical for the thermostability of PtIDH2 and that of marine psychrophilic bacterial type III NADP-IDHs are highly conservative, and suggests that there may be a close association between the two types of IDHs.

To dissect in-depth the evolutionary relationship between the novel eukaryotic type III NADP-IDHs (PtIDH2 and its counterparts) and other IDHs, we reconstructed a phylogenetic tree for 60 type III subfamily IDHs (Supplementary Figure S4). The tree contained two subtypes, namely, monomeric NAD-IDHs and complex NADP-IDHs. PtIDH2 and its homologs were classified into the NADP-IDH subtype and cross-distributed with cyanobacterial and psychrophilic NADP-IDHs. Indeed, PtIDH2 shared high amino acid sequence identity (∼60%) with the monomeric NADP-IDHs from marine psychrophilic bacteria and cyanobacteria, some of which coexist with *P. tricornutum* in marine and polar ecosystems, suggesting that PtIDH2 may have originated *via* horizontal gene transfer. Interestingly, we have previously shown that PtIDH1, a type II homodimeric NAD-IDH from *P. tricornutum*, is also presumed to have arisen *via* horizontal gene transfer from bacteria (Huang et al., 2020). BLAST analysis showed that the newly classified eukaryotic type III NADP-IDHs all derive from marine algae. Notably, all these marine algae belong to the stramenopile lineage, a clade that originated from cyanobacteria, Bacteroidetes, or Gamma-proteobacteria and experienced primary and secondary plastid endosymbioses. A large number of bacterial genes have been introduced into the eukaryotic nuclear genome *via* horizontal gene transfer (HGT) (Hopes et al., 2015; Brodie et al., 2017). Most genes acquired through horizontal gene transfer are located in the organelles (such as plastids or mitochondria) in which they exert their functions. Amino acid sequence alignment and predictive analysis showed that the full-length PtIDH2 containing the N-terminal targeting sequence (signal and transit peptide; Figure 2A) is very likely localized in the plastid. These results provide further evidence that PtIDH2 likely arose *via* horizontal gene transfer.

In conclusion, we have reported for the first time a novel NADP^+^-specific type III IDH from the eukaryotic microalga *P. tricornutum* and performed a detailed characterization of its biochemical properties. Meanwhile, through substitutional mutations of three residues pivotal for coenzyme binding, we were able to completely switch the coenzyme specificityof PtIDH2 from NADP^+^ to NAD^+^. In addition, several key amino acid residues involved in the thermostability of PtIDH2 were also identified. However, further studies on the structure of PtIDH2 are required to determine how homotetramerization occurs between its four monomeric IDH-like subunits, as well as understand the catalytic, regulatory, and evolutionary mechanism associated with this novel type III NADP^+^-specific IDH.

## Data Availability

The original contributions presented in the study are included in the article and its Supplementary Material. Further inquiries can be directed to the corresponding authors.
