# Supplementary figures and images for: Biochemical and Phylogenetic Characterization of a Novel NADP+-Specific Isocitrate Dehydrogenase From the Marine Microalga Phaeodactylum tricornutum
Source: Front Mol Biosci. 2021 Jul 5;8:702083. doi: 10.3389/fmolb.2021.702083 (PMC8287583; doi:10.3389/fmolb.2021.702083)

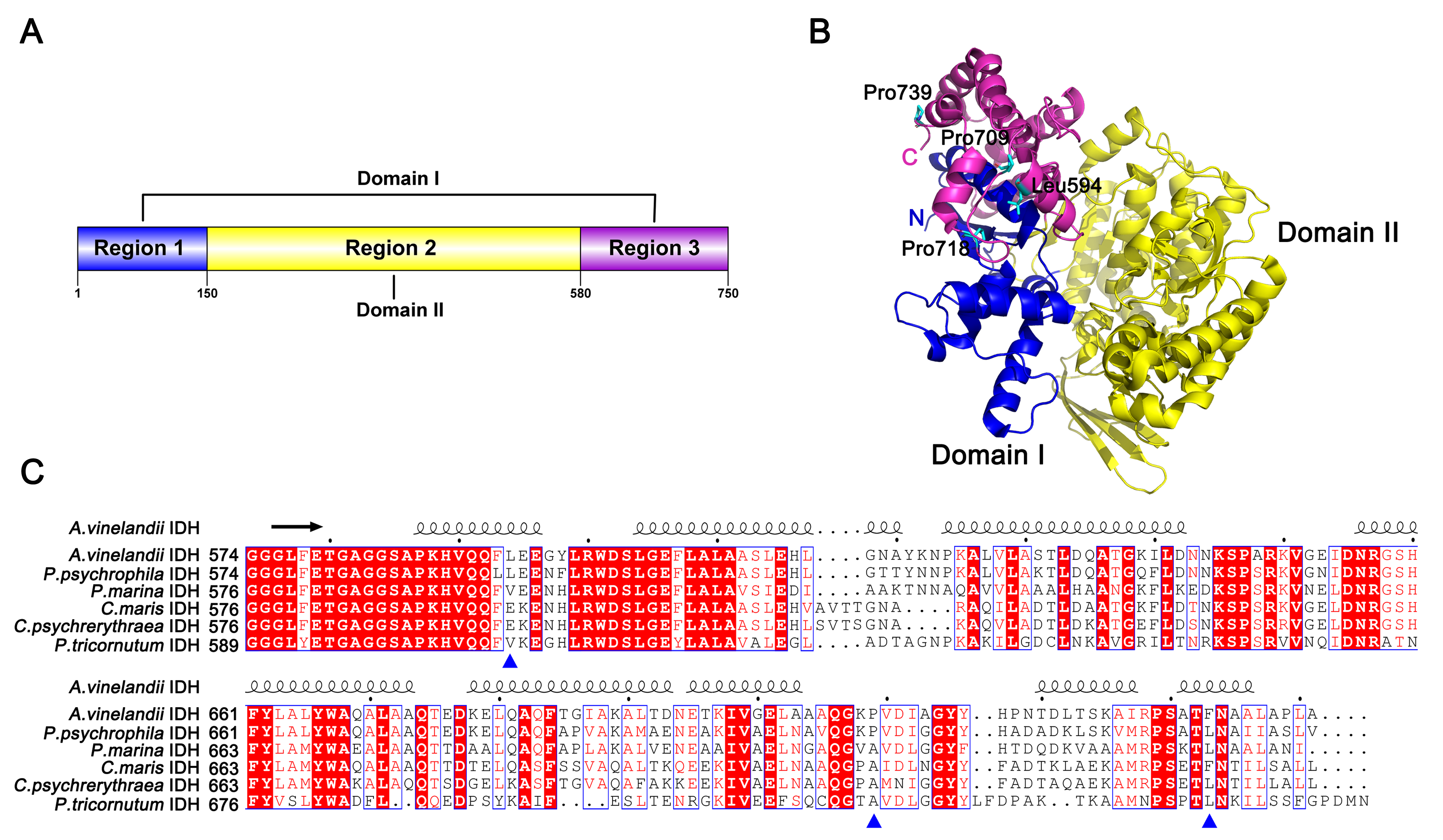

Supplement: Supplementary file 1 [file Image3.TIF]

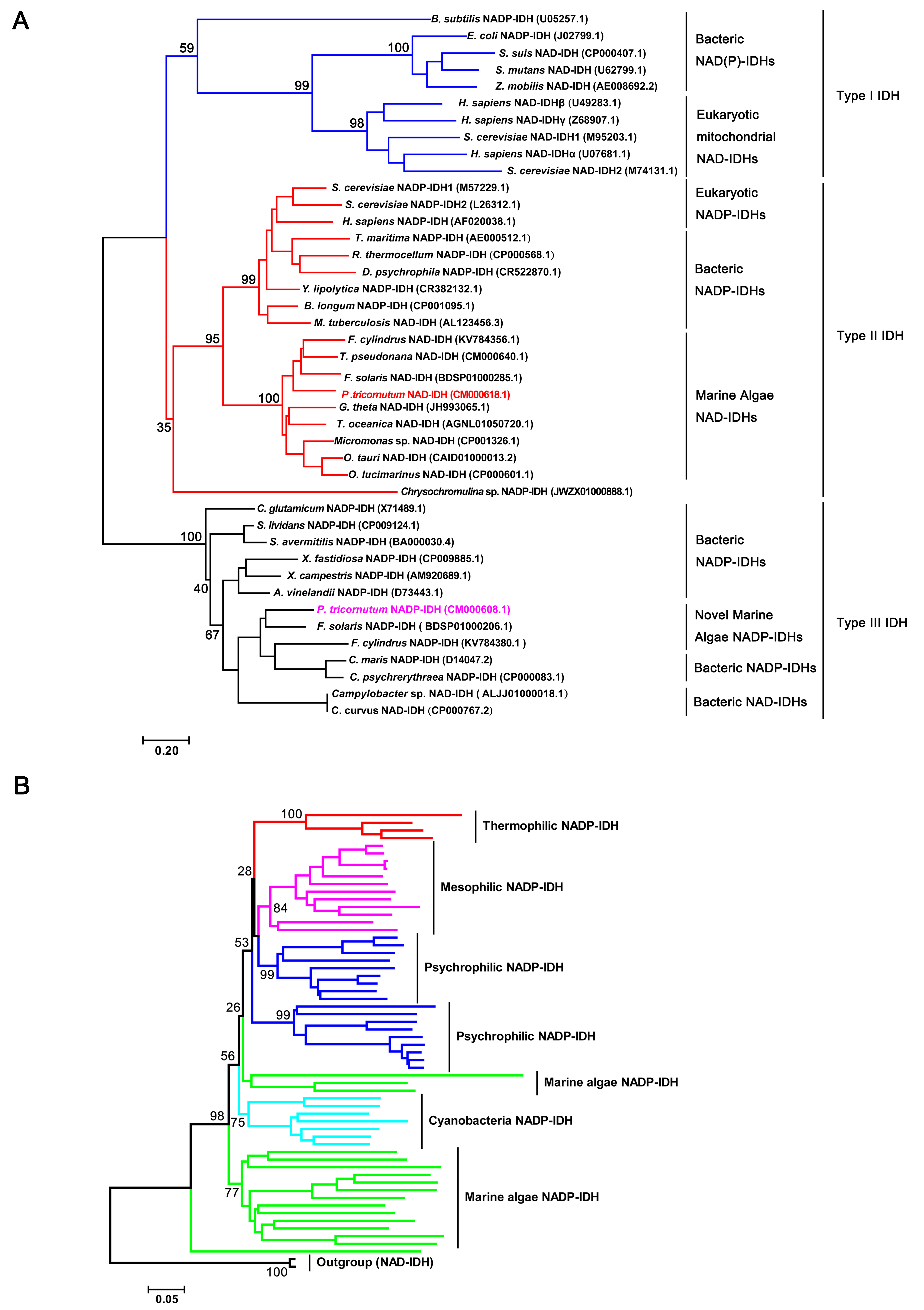

Supplement: Supplementary file 2 [file Image4.TIF]

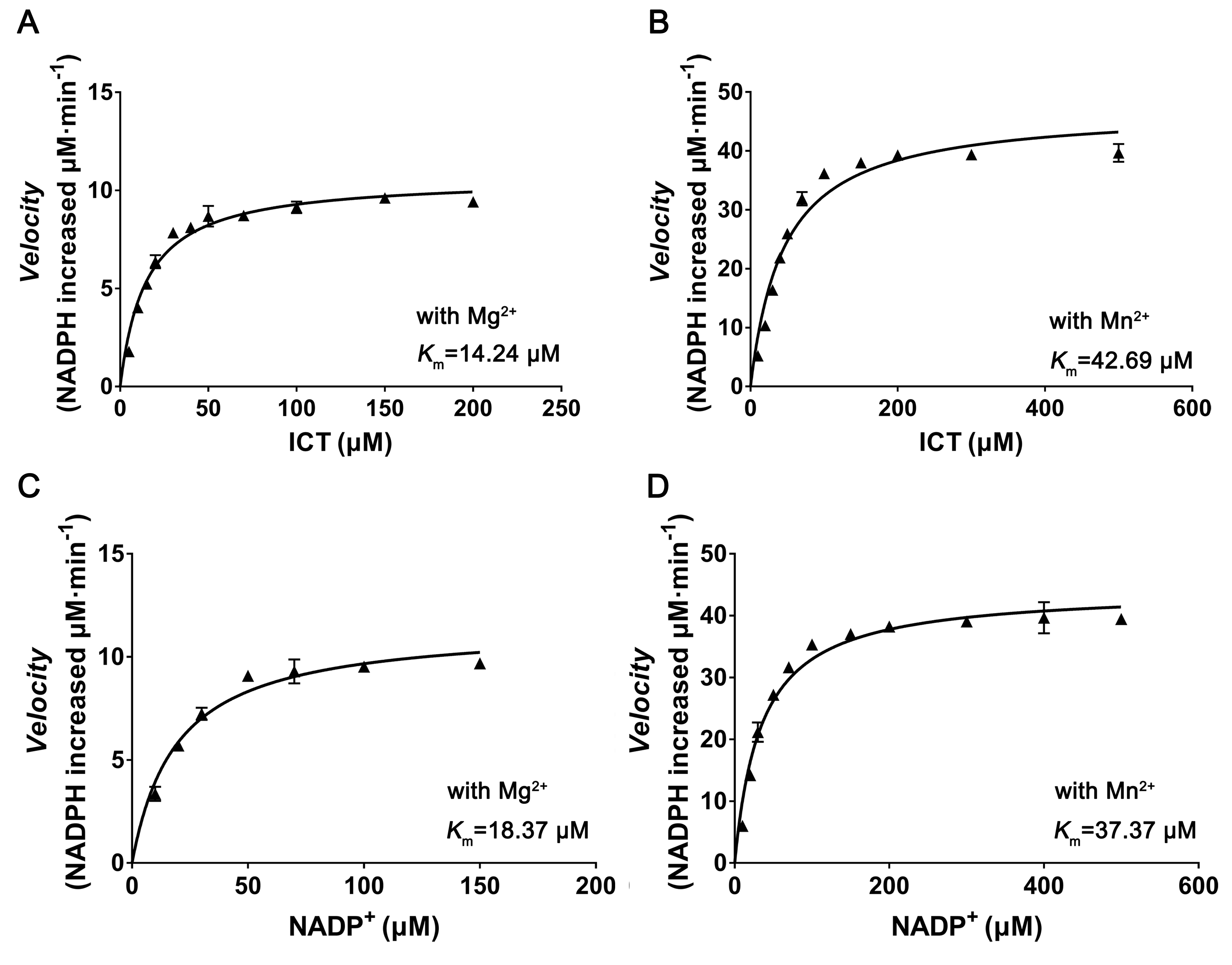

Supplement: Supplementary file 3 [file Image2.TIF]

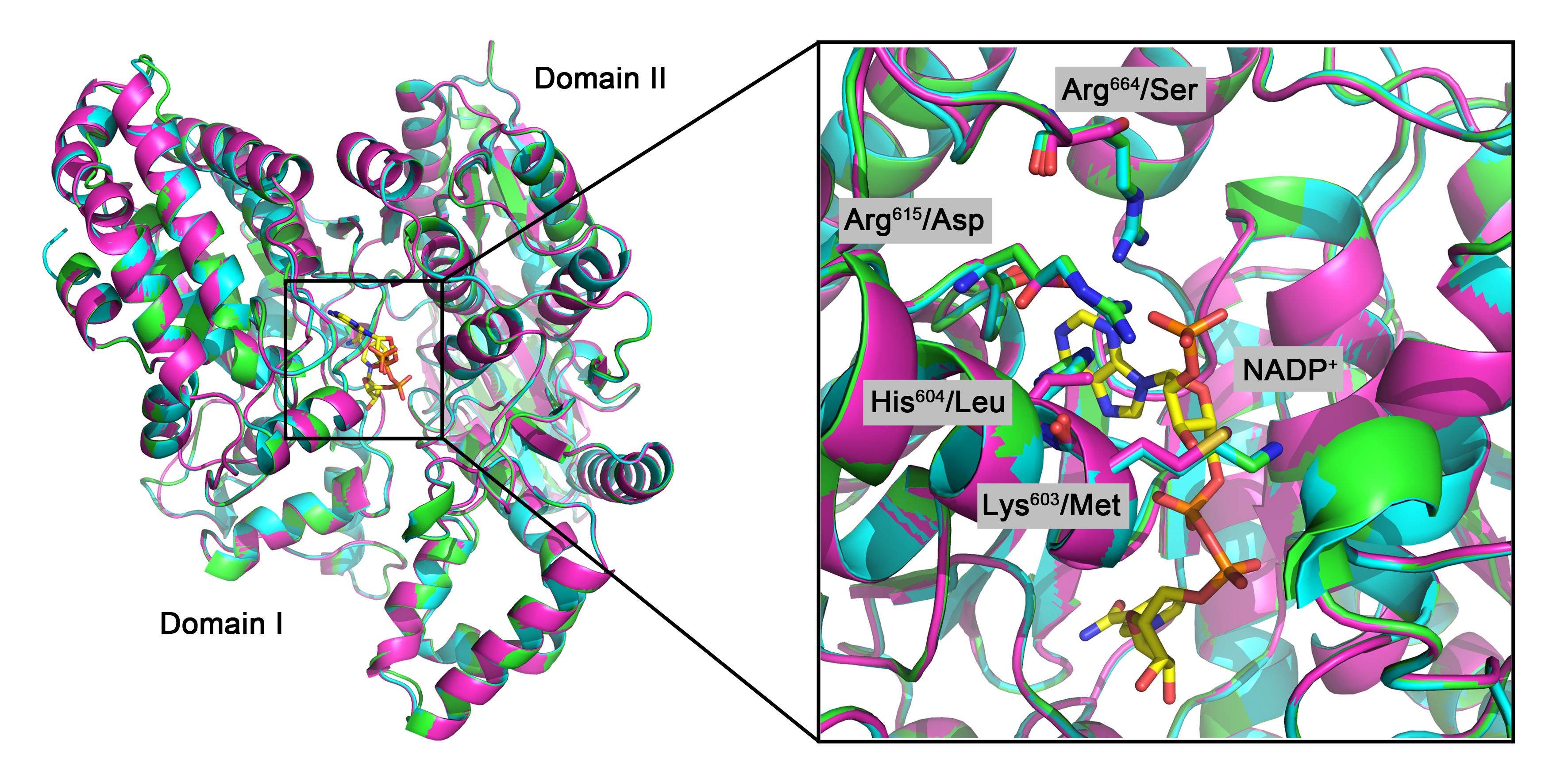

Supplement: Supplementary file 4 [file Image1.TIF]
