# Supplementary material for: Biochemical and Phylogenetic Characterization of a Novel NADP+-Specific Isocitrate Dehydrogenase From the Marine Microalga Phaeodactylum tricornutum
Source: Front Mol Biosci. 2021 Jul 5;8:702083. doi: 10.3389/fmolb.2021.702083 (PMC8287583; doi:10.3389/fmolb.2021.702083)
Supplement: Supplementary file 5 [file DataSheet1.PDF]

Supplementary Material

Table S1. Marine algae with the novel Type III NADP-IDHs.

Organism	UniProt Entry ID [a]	Clade [b]
Aureococcus anophagefferens	F0YF79	SAR (Heterokont)
Bigelowiella natans	JGI Genome: 57271	SAR (Rhizaria)
Cryptophyceae sp.	JGI Genome: 3630142	Cryptophyceae
Chrysochromulina parva	JGI Genome: 14991	Haptophyte
Chrysochromulina tobinii	A0A0M0K898	Haptophyte
Emiliania huxleyi	R1CPY1	Haptophyte
Fistulifera solaris	A0A1Z5KDP6	SAR (Heterokont)
Fragilariopsis cylindrus	A0A1E7ERJ3	SAR (Heterokont)
Guillardia theta	L1JYS0	Cryptophyceae
Phaeodactylum tricornutum	B7FVA8	SAR (Heterokont)
Pseudocohnilembus persalinus	A0A0V0QRC1	SAR (Alveolata)
Pseudo-nitzschia multistriata	A0A448Z4X6	SAR (Heterokont)
Sphaeroforma arctica	A0A0L0G9U0	SAR (Alveolata)
Symbiodinium microadriaticum	A0A1Q9ERZ3	SAR (Alveolata)
Thalassiosira oceanica	K0R3A1	SAR (Heterokont)
Thalassiosira pseudonana	B8BR03	SAR (Heterokont)
Micromonas commoda	C1FF99	Chlorophyta

[a] JGI Genome: https://genome.jgi.doe.gov/portal/.

[b] SAR: Stramenopiles/Alveolata/Rhizaria.

Table S2. Comparison of kinetic parameters between PtIDH2 and other IDHs.

Enzymes	NADP⁺			NAD⁺			Specificity	
	K_m (μM)	k_{cat} (s^{-1})	k_{cat}/K_m ($μM^{-1}·s^{-1}$) (A)	K_m (μM)	k_{cat} (s^{-1})	k_{cat}/K_m ($μM^{-1}·s^{-1}$) (B)	A/B	B/A
**Type III IDH**								
PtIDH2 (this study)	37.4	118.9	3.2	-	-	-		
A. vinelandii IDH (Watanabe et al., 2005)	5.8	92.6	15.8	ND	ND	ND		
C. glutamicum IDH (Chen and Yang, 2000)	4.0	87.0	22.0	19000	9.3	0.0005	44000	0.00002
S. avermitilis IDH (Wang et al., 2011)	4.98	58.2	11.7	6620	2.3	0.0004	29250	0.00003
A. baumannii IDH (Wang et al., 2018)	94	36.9	0.39	ND	ND	ND		
X. campestris IDH (Lv et al., 2016)	17.5	104.5	6	1100	18.3	0.017	353	0.0028
X. fastidiosa IDH (Lv et al., 2018)	1.0	96.5	96.5	2455	16.7	0.007	13786	0.00007
Campylobacter sp. IDH (Wang et al., 2015)	513.2	1.9	0.004	28.9	7.0	0.242	0.017	60.5
Engineered PtIDH2 (this study)	2704	1.01	0.0004	5825	7.67	0.0013	0.308	3.25
Engineered XcIDH (Lv et al., 2016)	1240	1.4	0.001	935	27.5	0.03	0.033	33
Engineered XfIDH (Lv et al., 2018)	959	8.9	0.009	5534	56.1	0.01	0.9	1.11
Engineered CaIDH (Wang et al., 2015)	11.4	4.2	0.368	493	9.5	0.02	18.4	0.054
**Type II IDH**								
B. longum IDH (Huang et al., 2016)	19.5	36.4	1.87	3584	11.7	0.003	623.3	0.0016
Y. lipolytica IDH (Li et al., 2013)	59	72	1.22	-	-	-		
O. tauri IDH (Tang et al., 2015)	3354	17	0.0051	226	59	0.261	0.0195	51.18
Engineered BlIDH (Huang et al., 2016)	324.1	0.307	0.00095	130	0.518	0.004	0.238	4.21
Engineered YlIDH (Li et al., 2013)	2410	4.24	0.00176	47000	0.38	0.000008	220	0.00455
Engineered OtIDH (Tang et al., 2015)	13.9	3.9	0.28	20652	80.9	0.0039	71.79	0.0139
**Type I IDH**								
E. coli IDH (Chen et al., 1995)	14	80.5	4.7	4700	3.22	0.0007	6714.3	0.00015
Z. mobilis IDH (Wang et al., 2012)	8200	14	0.0017	312	88	0.282	0.006	165.88
Engineered EcIDH (Chen et al., 1995)	5800	4.7	0.00081	99	16.2	0.164	0.0049	202.47
Engineered ZmIDH (Wang et al., 2012)	447	18.6	0.042	15000	10.7	0.0007	60	0.017

"-", no detectable activity. "ND", not mentioned.

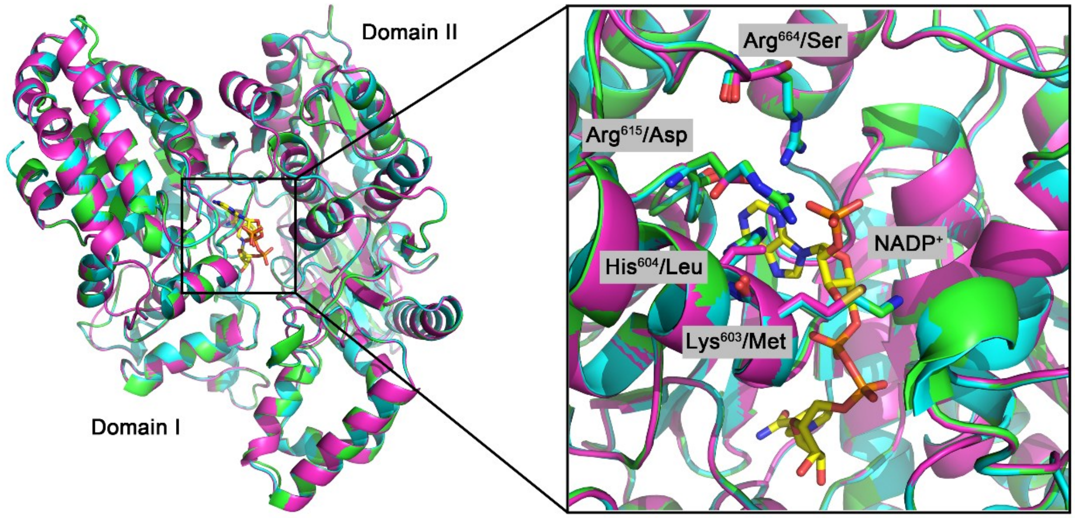

Figure S1. Modeling structure of mature PtIDH2. The left showing overlay of *A. vinelandii* NADP-IDH (green, PDB code 1J1W), model *Campylobacter* sp. NAD-IDH (purple) and model PtIDH2 (cyan). The right enlarged view displays the key residues which determine the coenzyme specificity by stick. Lys603, His604, Arg615 and Arg664 in PtIDH2 were targeted by mutagenesis.

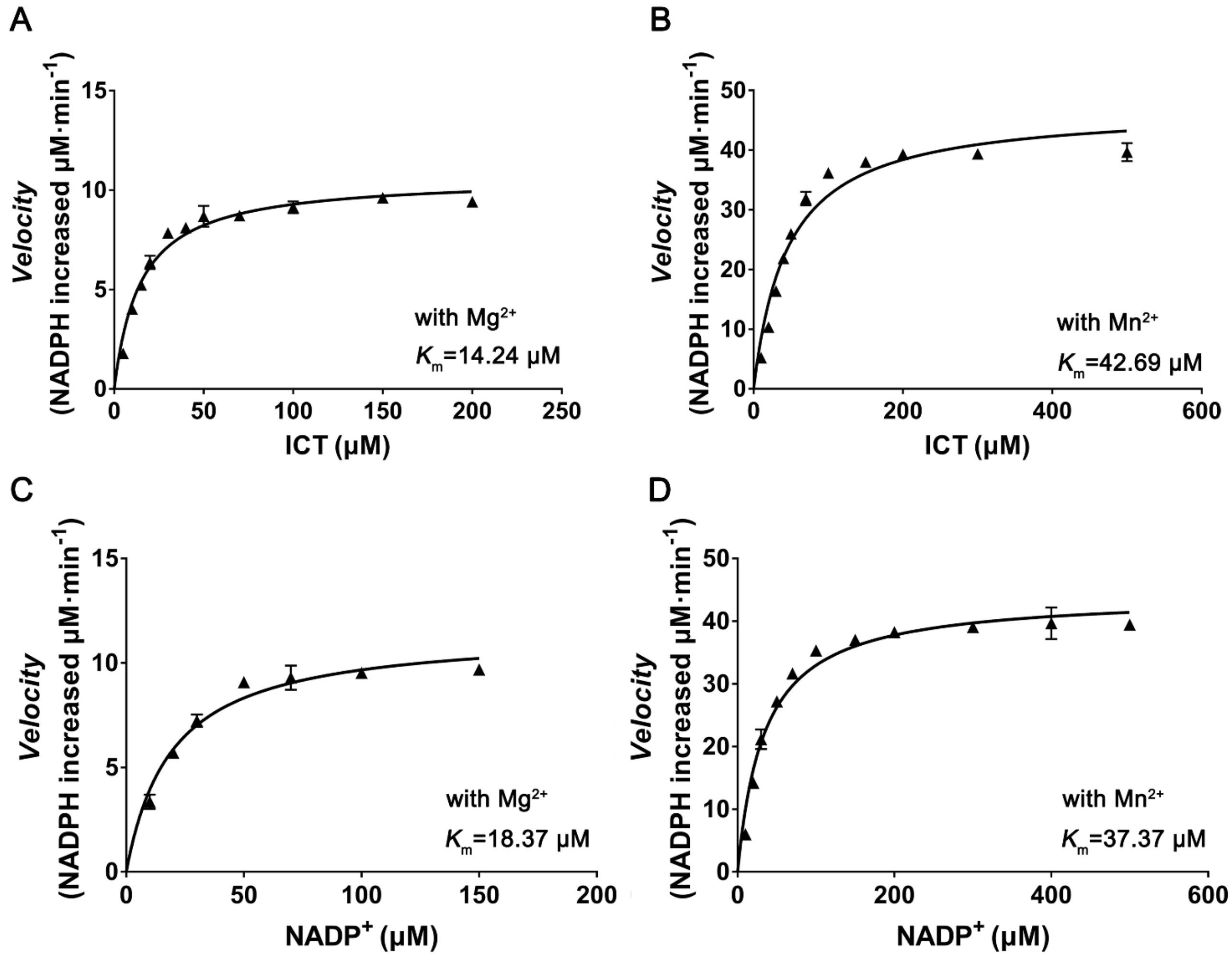

Figure S2. Kinetic analyses of the PtIDH2. The K_m values of PtIDH2 for ICT are 14.24 μM with Mg^{2+} **(A)** and 42.69 μM with Mn^{2+} **(B)**, respectively. The K_m values of PtIDH2 for $NADP^+$ are 18.37 μM with Mg^{2+} **(C)** and 37.37 μM with Mn^{2+} **(D)**, respectively.

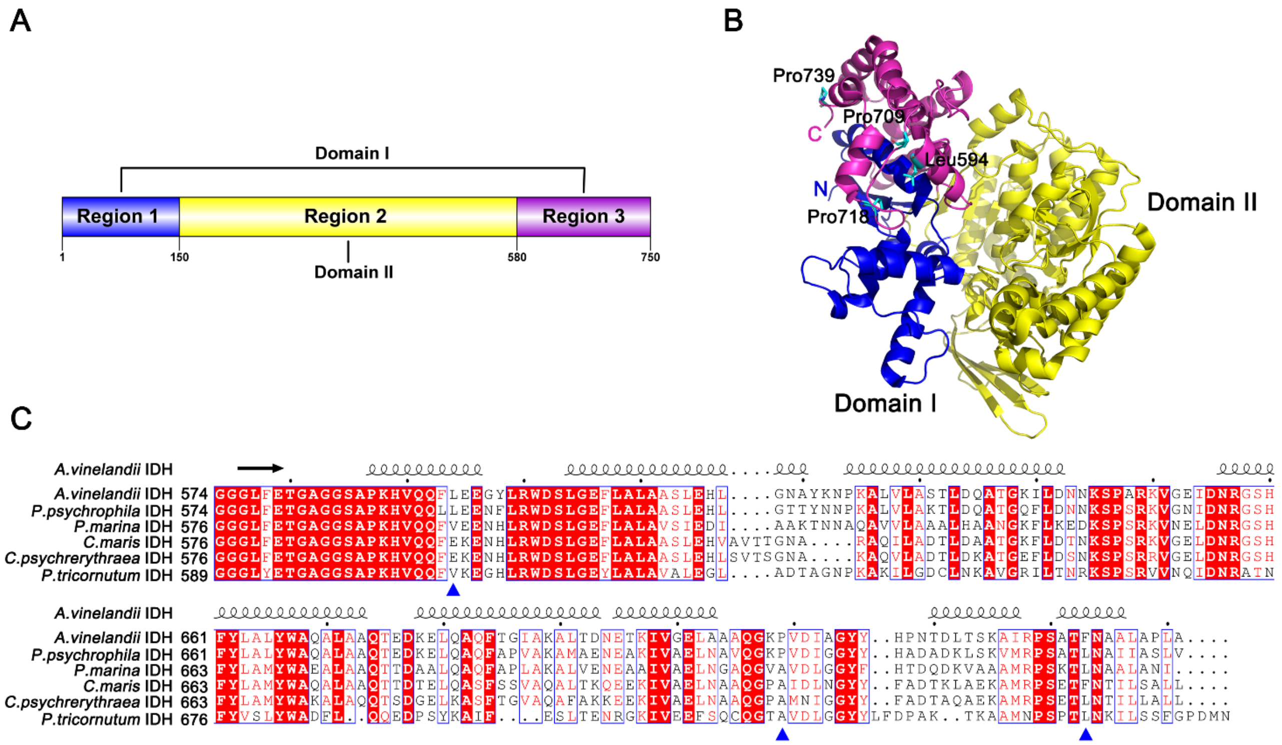

Figure S3. Amino acid sequences alignment between PtIDH2 and other momeric NADP-IDHs. **(A)** Diagram of structural motifs in monomeric IDH. **(B)** The overall structure of *A. vinelandii* IDH. The region I, II and III were indicated by blue, yellow and purple, respectively. **(C)** The alignment of amino acid sequence of monomeric IDHs from *A. vinelandii* IDH (UniProt ID: P16100, mesophilic IDH), *P. psychrophila* IDH (UniProt ID: C5NMM8, mesophilic IDH), *P. marina* IDH (UniProt ID: A0A0B6VJU5, psychrophilic IDH), *C. maris* IDH (UniProt ID: P41561, psychrophilic IDH), *C. psychrerythraea* IDH (UniProt ID: Q1XIQ9, psychrophilic IDH) and PtIDH2. The putative amino acid residues determinant in thermostability were noted by pentagram.

A

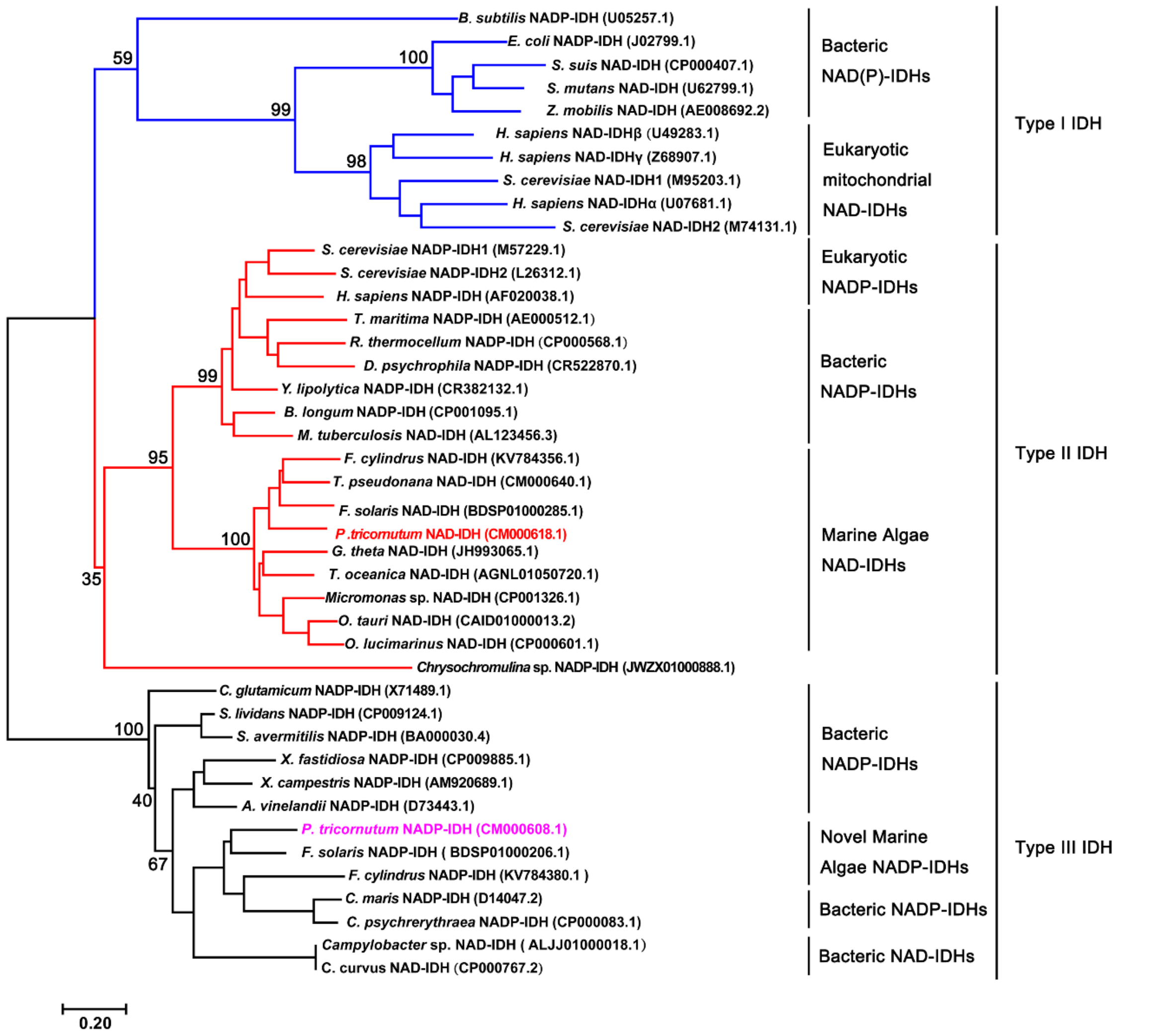

B. subtilis NADP-IDH (U05257.1)
E. coli NADP-IDH (J02799.1)
S. suis NAD-IDH (CP000407.1)
S. mutans NAD-IDH (U62799.1)
Z. mobilis NAD-IDH (AE008692.2)
H. sapiens NAD-IDHβ (U49283.1)
H. sapiens NAD-IDHγ (Z68907.1)
S. cerevisiae NAD-IDH1 (M95203.1)
H. sapiens NAD-IDHα (U07681.1)
S. cerevisiae NAD-IDH2 (M74131.1)
S. cerevisiae NADP-IDH1 (M57229.1)
S. cerevisiae NADP-IDH2 (L26312.1)
H. sapiens NADP-IDH (AF020038.1)
T. maritima NADP-IDH (AE000512.1)
R. thermocellum NADP-IDH (CP000568.1)
D. psychrophila NADP-IDH (CR522870.1)
Y. lipolytica NADP-IDH (CR382132.1)
B. longum NADP-IDH (CP001095.1)
M. tuberculosis NAD-IDH (AL123456.3)
F. cylindrus NAD-IDH (KV784356.1)
T. pseudonana NAD-IDH (CM000640.1)
F. solaris NAD-IDH (BDSP01000285.1)
P.tricornutum NAD-IDH (CM000618.1)
G. theta NAD-IDH (JH993065.1)
T. oceanica NAD-IDH (AGNL01050720.1)
Micromonas sp. NAD-IDH (CP001326.1)
O. tauri NAD-IDH (CAID01000013.2)
O. lucimarinus NAD-IDH (CP000601.1)
Chrysochromulina sp. NADP-IDH (JWZX01000888.1)
C. glutamicum NADP-IDH (X71489.1)
S. lividans NADP-IDH (CP009124.1)
S. avermitilis NADP-IDH (BA000030.4)
X. fastidiosa NADP-IDH (CP009885.1)
X. campestris NADP-IDH (AM920689.1)
A. vinelandii NADP-IDH (D73443.1)
P. tricornutum NADP-IDH (CM000608.1)
F. solaris NADP-IDH (BDSP01000206.1)
F. cylindrus NADP-IDH (KV784380.1)
C. maris NADP-IDH (D14047.2)
C. psychrerythraea NADP-IDH (CP000083.1)
Campylobacter sp. NAD-IDH (ALJJ01000018.1)
C. curvus NAD-IDH (CP000767.2)
59
100
99
98
99
95
100
35
100
40
67
Bacteric NAD(P)-IDHs
Eukaryotic mitochondrial NAD-IDHs
Eukaryotic NADP-IDHs
Bacteric NADP-IDHs
Marine Algae NAD-IDHs
Bacteric NADP-IDHs
Novel Marine Algae NADP-IDHs
Bacteric NADP-IDHs
Bacteric NAD-IDHs
Type I IDH
Type II IDH
Type III IDH
0.20

B

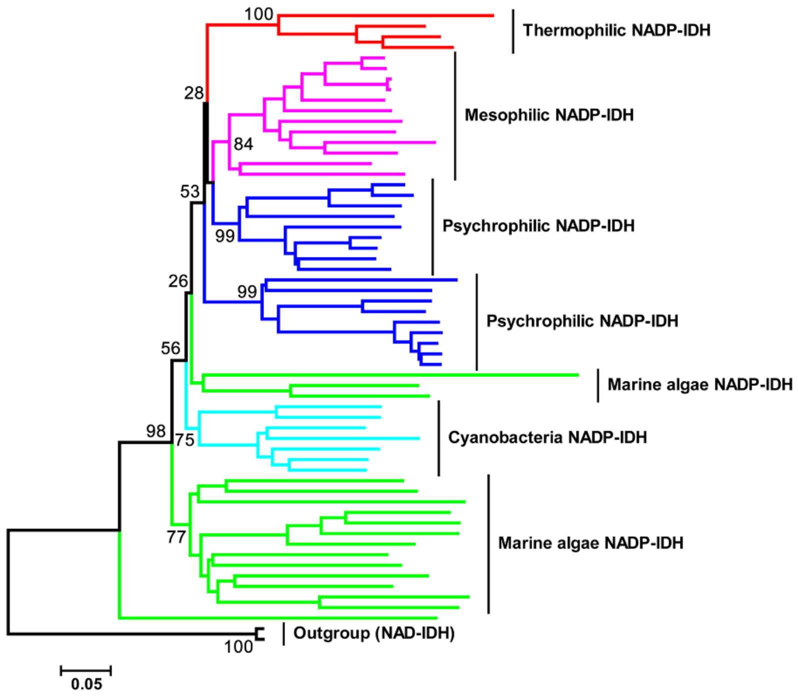

100
28
84
53
99
26
99
56
98
75
77
100
Thermophilic NADP-IDH
Mesophilic NADP-IDH
Psychrophilic NADP-IDH
Psychrophilic NADP-IDH
Marine algae NADP-IDH
Cyanobacteria NADP-IDH
Marine algae NADP-IDH
Outgroup (NAD-IDH)
0.05

Figure S4. Evolutionary relationships of PtIDH2 and other IDHs. **(A)** Phylogenetic analysis of the IDHs protein family. The analysis included the 42 IDH coding DNA sequences. A neighbor-joining tree with 1,000 bootstrap replicates was constructed using MEGA7. The GenBank numbers of all the IDHs are noted in parentheses. **(B)** Evolutionary relationships of 60 IDHs from marine algae and bacteria. A neighbor-joining tree with 1000 bootstrap replicates was created by using MEGA 7.0.

References

Chen, R.D., Greer, A., and Dean, A.M. (1995). A highly active decarboxylating dehydrogenase with rationally inverted coenzyme specificity. *Proc Natl Acad Sci U S A* 92, 11666–11670. doi: 10.1073/pnas.92.25.11666

Chen, R.D., and Yang, H. (2000). A highly specific monomeric isocitrate dehydrogenase from *Corynebacterium glutamicum*. *Arch Biochem Biophys* 383, 238–245. doi: 10.1006/abbi.2000.2082

Huang, S.P., Cheng, H.M., Wang, P., and Zhu, G.P. (2016). Biochemical characterization and complete conversion of coenzyme specificity of isocitrate dehydrogenase from *Bifidobacterium longum*. *Int J Mol Sci* 17, 296. doi: 10.3390/ijms17030296

Li, X., Wang, P., Ge, Y.D., Wang, W., Abbas, A., and Zhu, G.P. (2013). $NADP^+$-specific isocitrate dehydrogenase from oleaginous yeast *Yarrowia lipolytica* CLIB122: biochemical characterization and coenzyme sites evaluation. *Appl Biochem Biotechnol* 171, 403–416. doi: 10.1007/s12010-013-0373-1

Lv, C.Q., Wang, P., Wang, W.C., Su, R.R., Ge, Y.D., Zhu, Y.M., et al. (2016). Two isocitrate dehydrogenases from a plant pathogen *Xanthomonas campestris* pv. campestris 8004. Bioinformatic analysis, enzymatic characterization, and implication in virulence. *J Basic Microbiol* 56, 975–985. doi: 10.1002/jobm.201500648

Lv, P.P., Tang, W.G., Wang, P., Cao, Z.Y., and Zhu, G.P. (2018). Enzymatic characterization and functional implication of two structurally different isocitrate dehydrogenases from *Xylella fastidiosa*. *Biotechnol Appl Biochem* 65, 230–237. doi: 10.1002/bab.1560

Tang, W.G., Song, P., Cao, Z.Y., Wang, P., and Zhu, G.P. (2015). A unique homodimeric NAD^+-linked isocitrate dehydrogenase from the smallest autotrophic eukaryote *Ostreococcus tauri*. *FASEB J* 29, 2462–2472. doi: 10.1096/fj.14-257014

Wang, A., Cao, Z.Y., Wang, P., Liu, A.M., Pan, W., Wang, J., et al. (2011). Heteroexpression and characterization of a monomeric isocitrate dehydrogenase from the multicellular prokaryote *Streptomyces avermitilis* MA-4680. *Mol Biol Rep* 38, 3717–3724. doi: 10.1007/s11033-010-0486-3

Wang, P., Jin, M.M,, and Zhu, G.P. (2012). Biochemical and molecular characterization of NAD^+-dependent isocitrate dehydrogenase from the ethanologenic bacterium *Zymomonas mobilis*. *FEMS Microbiol Lett* 327, 134–141. doi: 10.1111/j.1574-6968.2011.02467.x

Wang, P., Lv, C.Q., and Zhu, G.P. (2015). Novel type II and monomeric NAD^+ specific isocitrate dehydrogenases: phylogenetic affinity, enzymatic characterization, and evolutionary implication. *Sci Rep* 5, 9150. doi: 10.1038/srep09150

Wang, P., Wu, Y.T., Liu, J., Song, P., Li, S., Zhou, X.X., et al. (2018). Crystal structure of the isocitrate dehydrogenase 2 from *Acinetobacter baumannii* (AbIDH2) reveals a novel dimeric structure with two monomeric IDH-like subunits. *Int J Mol Sci* 19, 1131. doi: 10.3390/ijms19041131

Watanabe, S., Yasutake, Y., Tanaka, I., and Takada, Y. (2005). Elucidation of stability determinants of cold-adapted monomeric isocitrate dehydrogenase from a psychrophilic bacterium, *Colwellia maris*, by construction of chimeric enzymes. *Microbiology* 151, 1083–1094. doi: 10.1099/mic.0.27667-0
